# Characterization of Elements Involved in Allosteric Light Regulation of Phosphodiesterase Activity by Comparison of Different Functional BlrP1 States^☆^

**DOI:** 10.1016/j.jmb.2013.11.018

**Published:** 2014-02-20

**Authors:** Andreas Winkler, Anikó Udvarhelyi, Elisabeth Hartmann, Jochen Reinstein, Andreas Menzel, Robert L. Shoeman, Ilme Schlichting

**Affiliations:** 1 Department of Biomolecular Mechanisms, Max Planck Institute for Medical Research, Jahnstrasse 29, 69120 Heidelberg, Germany; 2 Paul Scherrer Institut, 5232 Villigen PSI, Switzerland

**Keywords:** BLUF, Sensor of blue light using FAD, BlrP1, blue-light-regulated phosphodiesterase 1, c-di-GMP, cyclic dimeric GMP, EDTA, ethylenediaminetetraacetic acid, HDX, hydrogen–deuterium exchange, LED, light-emitting diode, MS, mass spectrometry, NMA, normal mode analysis, PDE, phosphodiesterase, SAXS, small-angle X-ray scattering, BLUF-photoreceptor, c-di-GMP, EAL, allostery, HDX

## Abstract

Bacteria have evolved dedicated signaling mechanisms that enable the integration of a range of environmental stimuli and the accordant modulation of metabolic pathways. One central signaling molecule in bacteria is the second messenger cyclic dimeric GMP (c-di-GMP). Complex regulatory mechanisms for modulating c-di-GMP concentrations have evolved, in line with its importance for maintaining bacterial fitness under changing environmental conditions. One interesting example in this context is the blue-light-regulated phosphodiesterase 1 (BlrP1) of *Klebsiella pneumoniae*. This covalently linked system of a sensor of blue light using FAD (BLUF) and an EAL phosphodiesterase domain orchestrates the light-dependent down-regulation of c-di-GMP levels. To reveal details of light-induced structural changes involved in EAL activity regulation, we extended previous crystallographic studies with hydrogen–deuterium exchange experiments and small-angle X-ray scattering analysis of different functional BlrP1 states. The combination of hydrogen–deuterium exchange and small-angle X-ray scattering allows the integration of local and global structural changes and provides an improved understanding of light signaling via an allosteric communication pathway between the BLUF and EAL domains. This model is supported by results from a mutational analysis of the EAL dimerization region and the analysis of metal-coordination effects of the EAL active site on the dark-state recovery kinetics of the BLUF domain. In combination with structural information from other EAL domains, the observed bidirectional communication points to a general mechanism of EAL activity regulation and suggests that a similar allosteric coupling is maintained in catalytically inactive EAL domains that retain a regulatory function.

## Highlights

• C-di-GMP is a central bacterial signaling molecule integrating environmental stimuli.
• BlrP1 enables the light-regulated degradation of c-di-GMP.
• Light-induced structural changes involve subtle quaternary structure rearrangements.
• Light signal integration involves allosteric signaling between BLUF and EAL.
• Analysis of different functional states reveals details of this communication pathway.

## Graphical abstract

**Figure:**
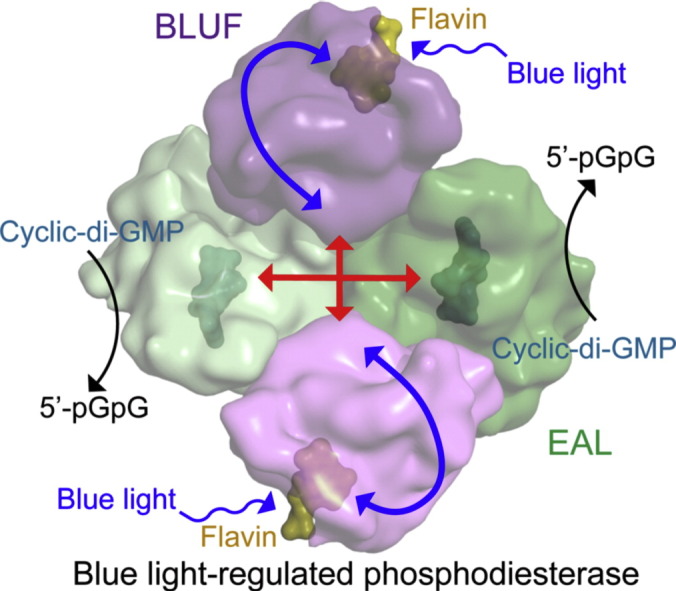

## Introduction

Allostery [1,2] is a widely used concept to describe various biomolecular processes ranging from protein dynamics, activation of membrane receptors or channels, chaperon function and virus assembly to allosteric enzymes (recently reviewed in Ref. [3]). The family of cyclic dimeric GMP (c-di-GMP)-interacting EAL domains [4] represents an interesting example. C-di-GMP was originally discovered as an allosteric activator of cellulose synthase in *Gluconacetobacter xylinus* [5] and has subsequently emerged as a central bacterial second messenger involved in regulating a wealth of cellular functions (reviewed in Refs. [6–8]). C-di-GMP's importance for bacterial homeostasis is reflected in the evolution of complex regulatory mechanisms that use c-di-GMP as an allosteric effector and in the modulation of its synthesis and degradation, which is controlled by various environmental sensor modules. EAL domains are involved in bacterial c-di-GMP signaling due to their phosphodiesterase (PDE) activity resulting in asymmetric cleavage of one substrate phosphodiester bond forming the linear 5′-pGpG product [4,9–13]. Additionally, however, sequence analysis [9,11] and mutational studies [13] revealed EAL subfamilies with mutations in otherwise highly conserved regions that lack PDE activity. It was suggested that such EAL domains can communicate the binding of c-di-GMP to a variety of effector domains via allosteric modulation [14–17]. Interestingly, EAL activity was also shown to be influenced in an allosteric manner upon GTP binding in GGDEF-EAL systems (reviewed in Ref. [18]) with degenerate GGDEF domains (reviewed in Ref. [19]) that lack their endogenous diguanylate cyclase activity [20,21]. Notably, EAL domains can also be regulated by a variety of other stimuli that are sensed by receiver (REC) domains [11]; Per-ARNT-Sim (PAS) domains [22–24]; domains found in cGMP-specific PDEs, adenylyl cyclases and FhlA (GAF) [25]; helix–turn–helix motifs [4]; phytochromes (PHY) [26]; or BLUF [10,12,15] modules.

The latter domain, acronym for sensor of blue light using FAD [27], enables the light-mediated control of different biological processes including gene transcription [28–30], phototaxis [31], adenylyl cyclase activity [32,33] and, as mentioned above, PDE reactivity in EAL domains. However, despite a great deal of information on these diverse systems, molecular and mechanistic details of light-induced changes in the vicinity of the flavin cofactor upon illumination are still under debate (recently summarized in Refs. [34] and [35]). Moreover, the conformational changes responsible for communicating the light signal to various effector domains regulated by BLUF are also not well understood. Therefore, the blue-light-regulated phosphodiesterase 1 [BlrP1; UniProt (UNP) ID: A6T8V8] from *Klebsiella pneumoniae*, which represents a covalently linked BLUF sensor and EAL effector system exhibiting a characteristic BLUF photocycle [36] and PDE activity [10], provides an intriguing system to improve our understanding of both allosteric regulation of EAL activity and light signaling by BLUF domains.

**Fig. 1:**
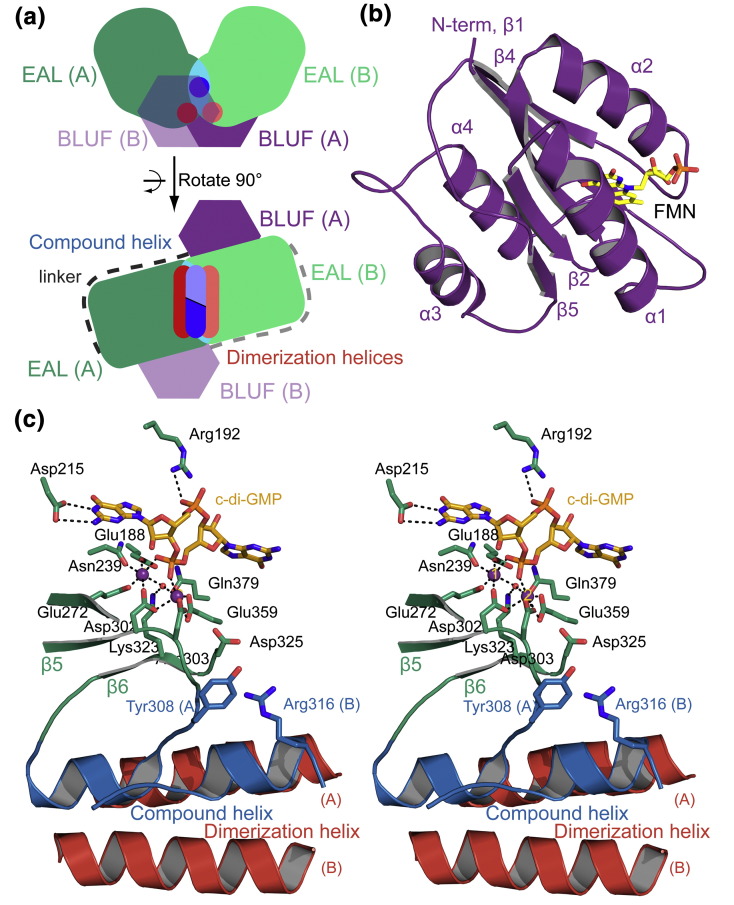
Overview of the *K*. *pneumoniae* BlrP1 structure. (a) Schematic representation of two different orientations of the BlrP1 structure reported previously [10]. The N-terminal BLUF domains are colored violet with protomer B in light color and transparent mode to show structural elements of the EAL domains in the background. EAL protomers A and B are colored dark and light green, respectively, and their overlap is in cyan. The compound helix formed by two short helices of protomers A and B in this region is illustrated in dark and light blue, respectively. The dimerization helices, one of each protomer, are colored dark and light red. (b) Cartoon representation of the BLUF domain to illustrate the arrangement of secondary structure elements (protomer B, PDB ID: 3GG0 [10]). The flavin cofactor is shown as yellow stick model. (c) Stereo view of the EAL active site and the dimerization region of BlrP1. The compound helix and the dimerization helices of the two protomers are colored according to (a). Regions involved in metal coordination and substrate binding are colored green, with important residues shown as stick models. The centrally coordinated metal centers are shown as purple spheres with labels for metal sites 1 and 2. The catalytic water molecule is represented by a small red sphere and c-di-GMP is shown as orange stick model. The conformation corresponds to the activated state of BlrP1 in the presence of manganese and high pH, PDB ID: 3GG0 [10].

Previous crystallographic studies of BlrP1 have revealed a dimeric arrangement of two EAL domains featuring an evolutionary conserved interface made up of two dimerization helices and one compound helix that is formed by two short helices provided by each protomer (Fig. 1a). Importantly, the light-sensing BLUF domains (Fig. 1b) are positioned close to this dimerization region [10]. PDE activity is also stimulated by an increase in pH that affects the coordination of the catalytically relevant metal ions in the active site. Based on this observation, a mechanism suggesting that structural changes induced by flavin excitation are passed on via the C-terminal BLUF capping helices to the EAL dimerization interface ultimately leading to a change of the EAL active-site geometry was proposed [10]. Despite the knowledge on structural details of dark-adapted BlrP1 and mechanistic insight into EAL regulation [10], there are still open questions related to structural aspects of the light-activated BlrP1 state and details of elements involved in the allosteric communication pathway.

Here we show for the first time structural details of the light-adapted BlrP1 state by integrating the analysis of global and local structural changes obtained from small-angle X-ray scattering (SAXS) studies and hydrogen–deuterium exchange (HDX) experiments analyzed by mass spectrometry (MS), respectively. We describe molecular details of EAL activity regulation by the BLUF sensor, providing evidence for an allosteric bidirectional communication between the flavin environment and the metal coordination in the active site of EAL. The compound helix, positioned at the EAL dimerization interface, plays a key role in this regulation, which is supported by complementary mutational and functional studies. In addition, SAXS experiments and normal mode analysis (NMA) suggest inter-domain rearrangements in BlrP1 that appear to be functionally conserved in other EAL dimers. In combination, our data provide new insight into molecular details involved in light sensing by BLUF domains and into regulatory aspects of EAL activity.

## Results

### The EAL dimer interface is influenced by substrate and calcium binding

**Fig. 2:**
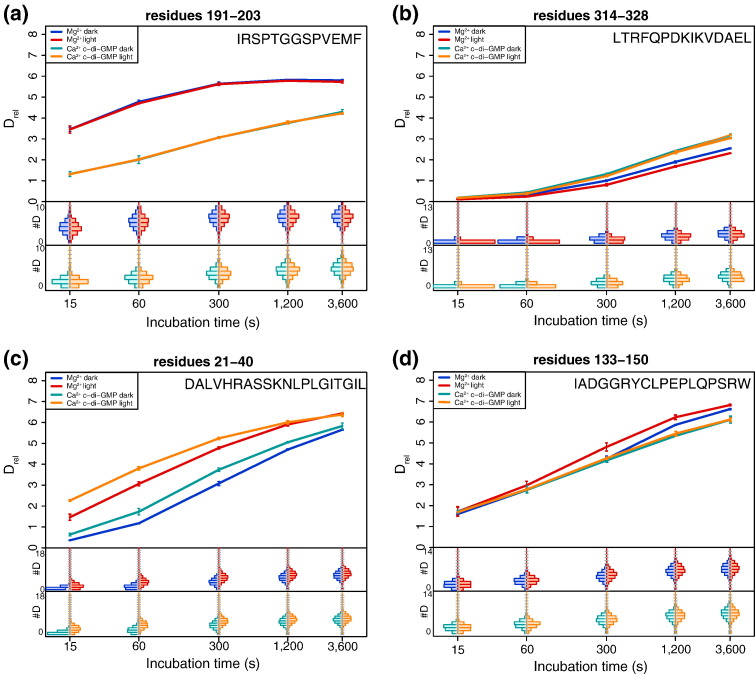
Deuterium incorporation plots of BlrP1 regions at four different experimental conditions addressed by HDX–MS. Labeling time dependence of relative deuterium incorporation of peptides indicated on top and the upper right corner of each panel are shown for Mg_d_, Mg_l_, Cc_d_ and Cc_l_ in blue, red, green and orange, respectively. The estimated abundance distribution of individual deuterated species is presented in the lower sub-panels on a scale from undeuterated to all exchangeable amides deuterated. (a) EAL region involved in substrate binding via Arg192 to one c-di-GMP phosphodiester bridge. (b) EAL element extending from the compound helix end to β6_E_ including conserved residues involved in, for example, water activation (Lys323). (c) α1-β2_B_ region of the BLUF domain responding to both light activation and metal and substrate binding. (d) Loop α3-α4_B_ in the BLUF capping helix region positioned in proximity to the EAL dimerization region. *D*_rel_ values are shown as the mean of three independent measurements with error bars corresponding to their standard deviation.

**Fig. 3:**
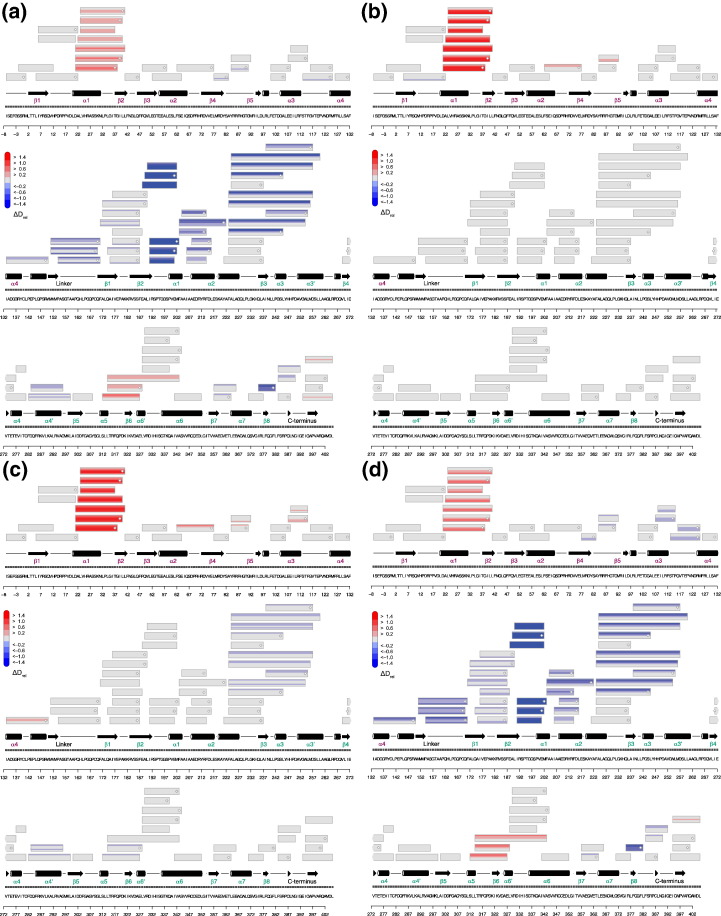
Overview of HDX experiments. Each box reflects one peptide and contains five different colors for the incubation times of 15, 60, 300, 1200 and 3600 s (bottom up), respectively. Individual colors correspond to the difference in relative deuteration (Δ*D*_rel_) of two compared states according to the legend on the left. MS/MS confirmed peptides are marked with diamonds and arrowheads at box termini indicate continuation of the peptide in the previous or following line. Secondary structure elements are taken from DSSP (*D*efine *S*econdary *S*tructure of *P*roteins) analysis of BlrP1, PDB ID: 3GG0 [10]. Numbering corresponds to the wild-type protein (UNP ID: A6T8V8) and negative values originate from the purification tag [10]. Secondary structure elements are indicated above the sequence and their labels are colored purple and green for BLUF and EAL domains, respectively. Zooming in on the electronic version allows viewing full details of all comparisons. (a) Cc_d_–Mg_d_. (b) Cc_l_–Cc_d_. (c) Mg_l_–Mg_d_. (d) Cc_l_–Mg_l_. Animations of the time course of Δ*D*_rel_ for all four comparisons with corresponding coloration of the BlrP1 crystal structure are shown in Movies S1–S4.

**Fig. 4:**
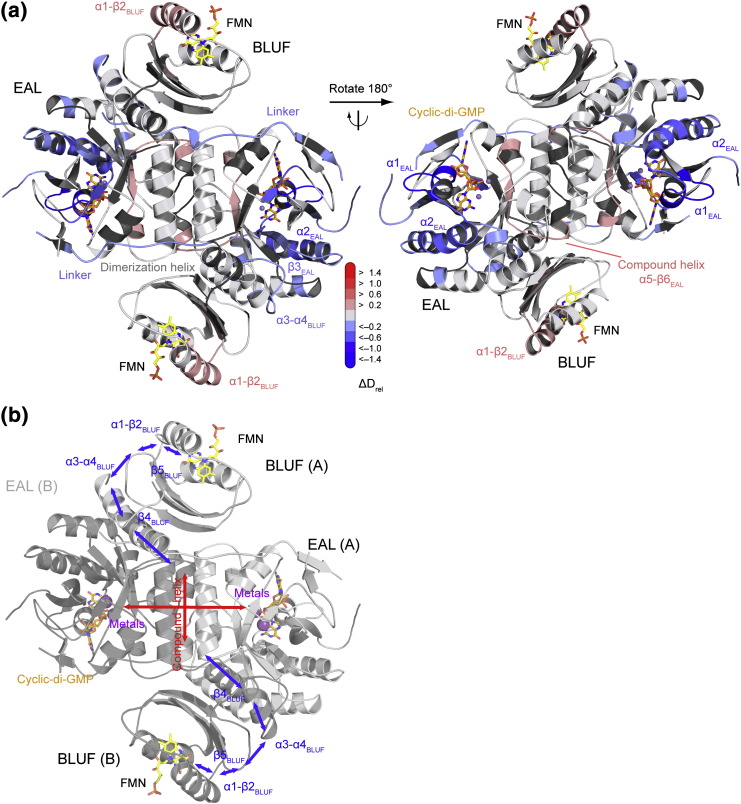
Summary of structural elements of BlrP1 involved in the light regulation of EAL activity. (a) BlrP1 structure colored according to changes in deuterium incorporation. A single time point (5 min) of the Cc_l_–Mg_l_ comparison is shown to illustrate the structural arrangement of BlrP1 elements involved in inter-domain communication based on PDB ID: 3GG0 [10]. Colors correspond to the differences in *D*_rel_ according to the bar legend. Red colors reflect an increased deuterium uptake upon substrate binding and calcium coordination, while blue colors indicate a stabilization of structural elements. Flavin mononucleotide (FMN) and c-di-GMP are shown as yellow and orange stick models, respectively, and metal ions as purple spheres. (b) Proposed allosteric signaling pathway between the BLUF and EAL domains. Based on the changes in deuterium incorporation in different functional states, a coupling of structural elements that supports the highlighted pathway was observed (see Discussion). BLUF-specific interactions are shown in blue and range from the place of photon absorption at the flavin cofactor (yellow sticks) to α3-α4_B_ and the C-terminal part of the β4_B_ that interact with the dimerization region of the EAL domains. The central role of this dimerization element is illustrated by the red arrows that indicate the coupling with the BLUF domains and the signaling to the EAL active sites where c-di-GMP (orange stick model) is bound. Cartoon representations of protomers A and B are colored light and dark gray, respectively.

As shown previously, c-di-GMP hydrolysis in BlrP1 is affected by changes in pH, by binding of divalent metal ions to the active site and by blue-light illumination [10]. It was proposed that absorption of a blue photon by the BLUF domain eventually results in changes in coordination of two metal ions that are critical for EAL activity (Fig. 1c). To obtain a better understanding of which structural elements are involved in the regulation of PDE activity, we probed four functionally relevant states of BlrP1 by HDX–MS: the substrate-free states of dark- and light-adapted BlrP1 in the presence of magnesium (Mg_d_ and Mg_l_, respectively; i.e., Magnesium_dark_ and Magnesium_light_); in addition, we addressed the influence of substrate binding by forming the inhibited EAL–Ca^2 +^–c-di-GMP complex also under dark and blue-light conditions (Cc_d_ and Cc_l_, respectively; i.e., Calcium–c-di-GMP_dark_ and Calcium–c-di-GMP_light_). Representative deuterium incorporation plots are shown in Fig. 2, and differences in relative deuterium levels (Δ*D*_rel_) for all assigned peptides in various states are summarized in Fig. 3. The localization of the most important structural elements in the quaternary BlrP1 assembly is illustrated in Fig. 4, and close-up views of important regions are provided in Fig. S1. Full details of all evaluated peptides are provided in Figs. S2–S5.

We initially compared Cc_d_ to Mg_d_ (Fig. 3a) to identify structural features that are affected by substrate and calcium binding in the dark. As expected, this comparison shows several elements that, based on the available crystal structures [10], are involved in substrate binding. The region containing the conserved Arg192, which is important for coordination of c-di-GMP via one of its phosphodiester bridges (Fig. 1c), shows the most pronounced stabilization (Fig. 2a). In the crystal structure, this loop region provides several residues contacting c-di-GMP while only few interactions are observed with the rest of the protein, thus explaining the pronounced protection of deuterium incorporation upon substrate binding (Fig. S1a). Additional elements stabilized by c-di-GMP binding involve residues Asp215, Gln379 and Asn239, all of which show a high degree of conservation among active EAL domains and are directly involved in substrate binding as judged from the crystal structure (Fig. 1c and Fig. S1a). Interestingly, one structural element shows an increase in deuterium incorporation deviating from the expected stabilization due to the presence of c-di-GMP (Fig. 2b). This element corresponds to the compound helix and the subsequent β-strand (β6_E_, subscript E or B indicates the secondary structure elements of the EAL or BLUF domains, respectively) projecting into the active site (Fig. S1b) without interacting directly with c-di-GMP. However, β6_E_ includes the conserved residues Asp325 and Lys323, of which Lys323 was previously proposed to be involved in orientation and activation of the catalytically active water molecule [10,13]. Considering the crystal structure of BlrP1 in its metal-free form (PDB ID: 3GFY [10]) and the disorder of the EAL dimerization region induced by the absence of the metal ions [10], it is suggestive that the observed decrease in HDX protection of the compound helix is due to the differences in the metal coordination (calcium instead of magnesium in Cc_d_ and Mg_d_, respectively).

Interestingly, substrate and calcium binding in the dark affected regions not only in the EAL domain but also in the BLUF-EAL linker and the α1-β2_B_ element (Fig. 2c and Fig. S1c). The latter region includes Asn31 that interacts with N3 and C4=O of the isoalloxazine ring system and close by residues, such as Arg26 and Lys30, interacting with the ribitylphosphate chain of the flavin cofactor. Since chemical shift perturbations upon illumination of the isolated BlrP1 BLUF domain [37] and NMR and HDX–MS studies of other BLUF proteins [30,38] showed that the α1-β2_B_ element is affected by blue-light activation, our data suggest a bidirectional communication between the BLUF and EAL domains. While the effect of flavin excitation on EAL activity in BlrP1 is well established [10], so far, no influence of ligands bound to an effector domain on the environment of the flavin cofactor has been described for any BLUF protein. Such a coupling of elements involved in substrate and metal coordination with the flavin environment indicates an allosteric signaling mechanism.

### Illumination affects EAL elements in the absence of calcium and substrate

In order to test the involvement of the α1-β2_B_ region also in the light response of full-length BlrP1, we first compared Cc_l_ to Cc_d_ (Fig. 3b) again because of the structural information available for substrate-bound, inhibited BlrP1. Indeed, the most pronounced light-induced changes affected peptides of the α1-β2_B_ region and evaluation of overlapping peptides suggests that the pronounced destabilization is most likely due to a reduction of α1_B_ stability upon blue-light illumination. Additional elements of the BLUF domain affected by illumination include β4_B_ that interacts with the EAL domain and β5_B_ that contains functionally relevant amino acids such as Met92. The latter, strictly conserved BLUF residue is especially interesting since two overlapping peptides in the β4-β5_B_ region allow detailed information of this residue to be extracted from HDX experiments (Fig. S6). Analysis of the overlap showed that the Met92 amide proton forms a relatively stable hydrogen bond in the dark that is significantly destabilized in the presence of blue light. In terms of the dark-adapted crystal structures, this can be rationalized by the hydrogen bond between the hydroxyl group of Ser28 and the Met92 amide group (cf. Fig. S1c; O–N distances of 3.2 and 2.7 Å for chains A and B of PDB ID: 3GFX, respectively). Importantly, Ser28 is also part of the abovementioned α1_B_ region that is pronouncedly destabilized upon illumination suggesting a correlation between the two structural elements. The destabilization of β4_B_ (residues 62–77; Fig. 3b) involves relatively slowly exchanging amides, where changes in deuteration are usually interpreted as changes in conformational dynamics. Therefore, illumination causes no distinct structural rearrangements in this region, which interacts with both the flavin cofactor and the EAL domain.

The lack of light-induced changes in the EAL domain complexed with calcium and c-di-GMP prompted the comparison of light- and dark-adapted states in substrate-free BlrP1 in the presence of magnesium (Mg_l_–Mg_d_). As shown in Fig. 3c, the same elements of the BLUF domain described above are affected. However, additional regions including the C-terminal BLUF helices, the linker region to the EAL domain and the PDE domain itself show characteristic changes in deuterium incorporation. Interestingly, similarly to α1-β2_B_ and β5_B_, a subtle destabilizing effect is observed for the C-terminal part of α3_B_ including residues of the loop to α4_B_ (residues 108–115; Fig. 3c). In the three-dimensional structure, this loop region is in direct contact with the previously mentioned β5_B_ region and the α4_B_ helix contacts the EAL dimerization interface (cf. Fig. S1d). Similar to these regions, the linker between BLUF and EAL (residues 133–150; Fig. 2d) also shows an increase in deuterium incorporation that was not observed in the Ca^2 +^-inhibited EAL domain upon illumination. Most importantly, however, light-induced changes observed in the Mg_l_–Mg_d_ comparison are transmitted to elements of the EAL domain that are involved in substrate binding and metal coordination (residues 238–255; Fig. 3c). This region contains the conserved Asn239 residue that is required for positioning of the metal 1 ion (Fig. 1c) [10]. Notably, this region is one of the structural elements that also shows altered deuterium incorporation upon substrate and calcium binding. In addition, a subtle effect of illumination is also observed for the compound helix described above (Fig. 2b). These, albeit moderate, changes in deuterium incorporation of additional elements identified in the Mg_l_–Mg_d_ comparison might explain light-induced changes in catalytic activity. However, this comparison does not provide detailed information of a specific chain of structural changes involved in transmission of the blue-light signal from the BLUF domain to the active site. Considering the Cc_l_–Cc_d_ comparison, it is also difficult to rationalize the contribution of metal coordination or substrate binding in preventing the changes of the compound helix and the active site observed in Mg_l_–Mg_d_. Importantly, both comparisons addressing the effect of BlrP1 illumination show no indications of an alteration of the oligomerization state of the characteristic EAL dimer, which would be expected to result in pronounced destabilization of the dimerization helix, the compound helix and elements involved in intermolecular BLUF-EAL contacts (Fig. 3b and c). The observation that light does not alter the oligomerization state is further supported by the solution scattering studies discussed below.

### Evolutionary conserved regions are involved in BLUF-EAL signaling

The observed changes in deuterium incorporation of the compound helix region upon both illumination and substrate/calcium binding suggest a central involvement of this element in the bidirectional communication of BLUF and EAL. Interestingly, this inter-subunit coupling is more pronounced in light-adapted BlrP1 (Cc_l_–Mg_l_ comparison; Fig. 3d). While the substrate- and calcium-induced effects on the EAL domain are similar to the Cc_d_–Mg_d_ comparison, the cross-talk with the BLUF domain is more pronounced in the presence of light. This is evident in increased changes in relative deuteration levels (Δ*D*_rel_) of previously identified elements such as the compound helix, the domain linker and the α1-β2_B_ region but, interestingly, also in additionally observed regions such as the loop between the BLUF capping helices (α3-α4_B_) and the C-terminal part of the β4_B_ element (Fig. 3d and Fig. S1d). Both regions contact the EAL domains near their dimerization and compound helices. Notably, the observed destabilization of α1-β2_B_ is additive as indicated by the enhanced destabilization due to calcium and substrate binding in the presence of blue light (Fig. 3d, residues 21–40).

In summary, the results of our HDX analysis reveal elements involved in light regulation and metal coordination of the EAL domain and the combination of all comparisons enables the mapping of a potential signaling pathway between the sensor and effector domains (Fig. 4b). They provide molecular details of regions involved in inter-domain communication supporting the critical role of previously proposed elements important for signal transduction. This includes the α1-β2_B_, β4_B_, β5_B_ and α3-α4_B_ (capping helices) regions and elements close to the compound helix involved in substrate binding and metal coordination in the EAL domain (Fig. 3d). In combination with the observation that c-di-GMP binding does not induce significant structural rearrangements of the EAL domain [16,24], the observed light- and metal-induced changes of the BLUF-EAL linker region and of interface elements involving the BLUF C-terminus and the EAL dimerization interface suggest inter-domain rearrangements accompanying the coupling of receptor and effector domains.

**Fig. 5:**
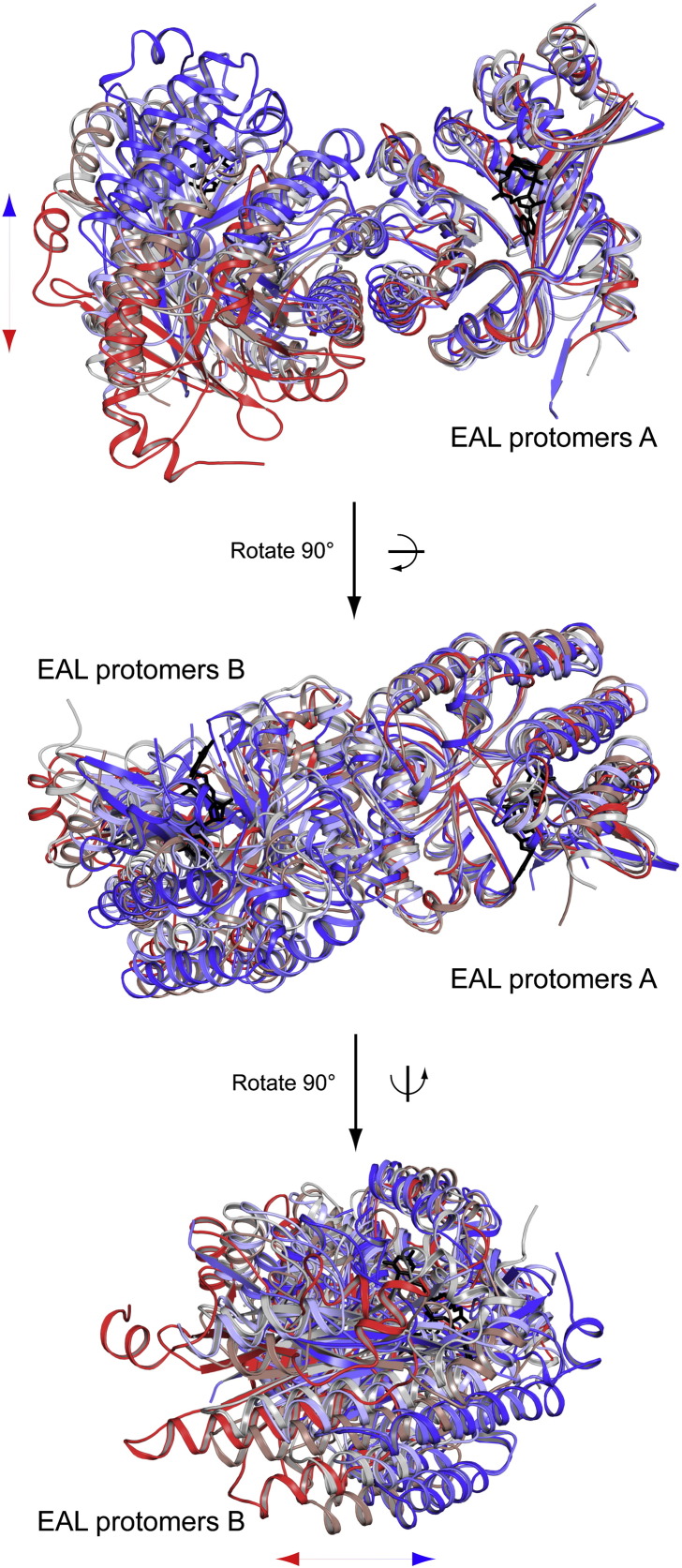
Structural superposition of various EAL domain dimers. Structural alignments with respect to protomers A of the characteristic EAL dimers of YkuI (UNP ID: O35014) [24], BlrP1 [10], *Td*EAL (UNP ID: Q3SJE6) [13], *Cc*EAL (UNP ID: Q9A310 and PDB ID: 3U2E; unpublished), RocR (UNP ID: Q9HX69) [40] and DosP (UNP ID: P76129) [39] are shown in cartoon representation. The orientations of the upper two panels correspond to those of Fig. 1a and colors reflect the amplitude of the clam-shell opening of the EAL dimer. Dark blue corresponds to YkuI and the most pronounced closed state and red belongs to DosP with the largest opening. Transitions over blue, light blue, gray and light red correspond to BlrP1, *Td*EAL, *Cc*EAL and RocR, respectively. Representative c-di-GMP molecules of YkuI are shown as black stick models to indicate the substrate binding sites. The preferred orientation of EAL protomers along one trajectory and the similarity to normal mode movements (Fig. S7c) further support the functional relevance of the EAL dimer assembly.

To test this hypothesis, we performed SAXS studies of BlrP1 under conditions resembling Mg_d_ and Mg_l_ and used NMA for structural interpretation (Supplementary Data and Figs. S7 and S8). Importantly, the radial density distributions of BlrP1 in the dark and light states overlap at small scattering angles (Fig. S7d). This confirms that illumination does not result in changes of the oligomerization state, which is a prerequisite for interpreting the HDX data in terms of an allosteric signaling pathway. A closer comparison between the experimental data and the theoretical scattering curves calculated from the BlrP1 crystal structure and its computed normal modes suggested that the observed differences correspond to inter-domain rearrangements (cf. Supplementary Data). Interestingly, the structural difference between the dark-state BlrP1 dimer in solution and *in crystallo* corresponds to a clam-shell opening of the EAL domains (Fig. S7c). This structural movement resembles different EAL dimer arrangements observed in various EAL structures [10,13,24,39,40] (Fig. 5), which implies a functional relevance of the opening–closing movement of the EAL dimer. The light-induced differences in the scattering curves can be explained by a twisting motion that results in a subtle reorientation of the BLUF domains relative to the EAL domains, which also affects the opening and closing of the EAL dimer (Fig. S7f). Considering the coupling of the two inter-domain rearrangements, these are the global structural changes that are responsible for light regulation of EAL activity. Importantly, the contact sites between the BLUF and EAL domains that communicate the quaternary rearrangements between the two domains (Fig. S7f) correspond to elements identified by the HDX measurements. An analysis of the evolutionary conservation of BlrP1 residues further highlights the functional relevance of the BLUF-EAL interface involving the β4_B_ and capping helix (α3-α4_B_) regions of BLUF and elements close to the compound helix of EAL (Fig. S9). These regions show a comparable evolutionary conservation to residues lining the c-di-GMP binding pocket and the EAL dimerization interface, which further supports the functional relevance of the identified structural elements in conformational coupling between the EAL and BLUF domains.

### The compound helix environment is involved in inter-domain communication

Based on the observation of a bidirectional communication between BLUF and EAL described above, we tested the influence of metal coordination of the EAL domain on the dark-state recovery kinetics of the BLUF photocycle. As summarized in Table 1, we addressed catalytically active forms of full-length BlrP1 in the presence of magnesium or manganese and inactive calcium-bound or metal-free states in the presence or absence of the substrate c-di-GMP. In addition, we included the BlrP1 BLUF domain as a control to probe any potential metal effect on the dark-state recovery kinetics of the isolated photoreceptor domain and to dissect the additional influence of the EAL domain. While no significant metal- or protein-construct-dependent differences in the characteristic BLUF dark-state spectrum or the ~ 10-nm red-shifted spectrum of the light-activated state of BlrP1 were observed (Fig. S10), the dark-state recovery rates significantly differed for the various conditions (Table 1).

The influence of the EAL domain alone can be inferred from the ethylenediaminetetraacetic acid (EDTA)-treated full-length sample *versus* that of the isolated BLUF domain. Similar to recent reports for a non-covalently linked BLUF-EAL system from *Rhodopseudomonas palustris* [12], we observed an effect of the EAL domain on the BLUF photocycle supporting a cross-talk between the two domains. EDTA chelation successfully removes metal ions as indicated by the consistent dark-state recovery kinetics with two EDTA-treated BlrP1 variants that otherwise show different metal-induced effects (Table 1, Y308F and R316M; cf. Fig. 1c and details below). Interestingly, the observed slower dark-state recovery in full-length BlrP1 compared to the isolated BLUF domain is even more pronounced in the metal-activated states with magnesium or manganese bound to the EAL domain. Importantly, the influence of calcium, which inhibits EAL catalytic activity, is opposite to that of either magnesium or manganese. In fact, the dark-state recovery is accelerated and resembles that of the BLUF domain alone. This, however, does not indicate uncoupling between the two domains as reflected by the small but reproducible additional decrease in the lifetime of the red-shifted BLUF spectrum upon addition of c-di-GMP to either EDTA-treated or calcium-inhibited BlrP1 (Table 1). Importantly, the more pronounced effect of metal coordination compared to substrate binding observed in dark-state recovery experiments also indicates that the observed differences in deuterium incorporation for elements along the signaling pathway are dominated by metal coordination. Although the effects on the recovery kinetics are relatively small, they are highly accurate and the precision indicated by the standard deviation is also supported by control measurements using different wild-type BlrP1 batches in the presence of Mg^2 +^ or EDTA.

Importantly, the trends described here support the conclusions drawn from HDX–MS and SAXS measurements concerning the bidirectional communication between the light-sensing BLUF domain and the residues involved in metal and substrate coordination in the EAL domain. Therefore, the measurements of dark-state recoveries were used to additionally characterize the effect of perturbations in the EAL dimerization interface on PDE activity. Previously, it was observed that a variant containing two point mutations in this region (BlrP1 S309C S312C) shows significantly reduced enzymatic activity [10]. Since the catalytic activity of this variant is below the detection level, no detailed insight into the role of these amino acids for the light-activation pathway is possible. Therefore, we made two additional single point mutations in the loop regions preceding and following the compound helix, Y308F and R316M, respectively. Arg316 is involved in stabilizing the dimeric EAL arrangement by inter-protomer contacts with the Gly307 carbonyl group in the presence of c-di-GMP and manganese (PDB ID: 3GG0), whereas no such interaction is observed in the other BlrP1 structures [10]. The Tyr308 side chains of both monomers are located at the center of the compound helix, and they are positioned such that they can interfere with the Arg316–Gly307 interaction. In contrast to Arg316, Tyr308 only adopts a well-defined conformation in the low pH, Ca^2 +^–c-di-GMP structure (PDB ID: 3GFX [10]). The structural element containing Tyr308 is interesting also because it shows a high degree of asymmetry between the two protomers compared to the overall symmetric EAL dimer arrangement.

The enzymatic activity of Y308F and R316M variants was tested as described previously [10] and found to be reduced by a factor of ~ 9 and ~ 5, respectively (Table 2). Interestingly, however, the blue-light-induced ca 4-fold stimulation of EAL activity is retained by both variants. While the EDTA-treated samples of both variants show the same dark-state recovery kinetics as wild-type BlrP1, the distinct stabilization of the light-activated state due to magnesium binding is missing. The acceleration of the dark-state recovery induced by Ca^2 +^ coordination, however, is more pronounced than in the wild type, suggesting that either metal binding to the active site or structural consequences thereof are affected.

The results from dark-state recoveries and catalytic activity measurements support the conclusion that EAL activity is regulated via the EAL-EAL dimerization interface. As indicated by the dark-state recovery kinetics of two variants of this region, changes at the interface likely affect metal coordination in the active site and are coupled to the BLUF domain, where they influence the flavin cofactor environment, leading to either stabilization or destabilization of the light-activated state under conditions resembling Mg_l_ and Cc_l_, respectively. Especially the substitution of Tyr308 that is positioned at the contact site of the two short helices forming the compound helix has a pronounced effect on the PDE activity. This further confirms the central role of the dimeric EAL assembly for regulation of c-di-GMP hydrolysis and that the modulation of the quaternary arrangement is exploited by the light-sensing BLUF domain in an allosteric manner (Fig. 4b).

## Discussion

Our studies of the blue-light-regulated PDE BlrP1 provide new structural and functional insights into regulation of EAL domains with implications not only for allosteric control of PDE activity but also for control of other c-di-GMP responsive processes that are mediated by inactive EAL domains. In addition, the observed cross-talk of the EAL domains with the flavin-based BLUF photoreceptor domains presents new aspects of structural elements involved in blue-light sensing of BLUF domains.

In the context of BLUF signaling, open questions concern the mechanism of signal transduction to various effector domains (reviewed in Ref. [41]) and the controversial interpretation of molecular details of hydrogen bonding in the flavin binding pocket with implications for the photoactivation mechanism (summarized in Ref. [35]). While the spatial resolution of HDX–MS limits its use for addressing differences in the hydrogen bonding network of individual residues, HDX–MS has the advantage of probing light-induced changes in the context of full-length proteins providing both global and local structural information. The observed changes in deuterium uptake of BLUF elements are in good agreement with previous NMR studies of the isolated BlrP1 BLUF domain [37] and demonstrate the importance of the α1-β2_B_ region, the β4-β5_B_ loop, β5_B_ and the α3-α4_B_ loop in the C-terminal capping helices also for the holo protein. In addition, our experiments including the EAL domain in different functionally relevant states further suggest a light-induced signaling cascade that originates in the α1-β2_B_ region and β5_B_. Interestingly, α1-β2_B_ appears to be a central element of the blue-light response of BLUF domains as also observed in similar studies with AppA (UNP ID: Q53119) [30]. Depending on the metal-coordination state of the EAL domain, this structural change can be further communicated to the β4-β5_B_ and α3-α4_B_ loops and to β4_B_ that directly interact with elements of the EAL dimerization region. These observations reveal new molecular details of the initially proposed signaling pathway [10] and support the central role of the C-terminal capping helices in communicating the signal to the compound helix environment of the EAL dimer. This crucial role of the BLUF capping element was recently also shown for photoreceptor chimeras featuring capping helices from different BLUF systems [42] and HDX–MS studies on the prototypic BLUF member AppA in complex with its non-covalently linked effector PpsR [30]. However, given the markedly different structures of the capping helices (Fig. S11; Refs. [10,30,43] and [44]), these elements appear to have evolved as system-specific features that relay the light signal from the BLUF core to their corresponding effector regions.

With respect to signal transmission to EAL domains, it is interesting to note that the EAL conformation dominates the communication with the BLUF domain. In the EAL inhibited state with substrate and calcium present, light only triggers changes in the close vicinity of the flavin cofactor (α1-β2_B_ and β5_B_; cf. Cc_l_–Cc_d_). In contrast, substrate and calcium binding in the light-activated state (cf. Cc_l_–Mg_l_) shows that substrate binding and metal coordination induce changes all along the signaling pathway and even affect the α1-β2_B_ region. Illumination, however, also affects structural elements in the substrate binding site and the EAL dimerization region in the presence of magnesium and in the absence of c-di-GMP (cf. Mg_l_–Mg_d_). This bidirectional cross-talk of EAL and BLUF domains supports an allosteric communication pathway that involves the EAL dimer interface as central communication platform. Light signals from both BLUF domains are integrated at the conserved EAL dimerization region and communicated to the EAL active sites. Such an inter-domain coupling in the BlrP1 dimer satisfies allosteric concepts such as global dyad symmetry, and typically, communication in a co-operative dimeric assembly (Fig. 4b) results in the amplification of the light response [45]. A similar symmetry as observed for the BLUF-EAL couple in BlrP1 is also present in the crystal structure of YkuI that features a PAS-EAL couple [24]. The positioning of the individual modules appears to be very similar in both cases [10], and the BLUF and PAS domains are placed close to the EAL dimerization regions. Importantly, in both cases, the overall symmetry is governed by the evolutionary conserved EAL dimerization interface that is observed for all catalytically active EALs and some inactive domain structures [10,13,24,39,40]. A superposition of all structures with respect to protomer A shows that the major differences in positioning of the second EAL domain correspond to a specific opening–closing transition of the EAL dimer (Fig. 5) that also resembles the different BlrP1 conformations observed under different experimental conditions by SAXS in solution and by X-ray crystallography. Interestingly, the light-induced structural change also modulates this specific movement of the EAL domains. Therefore, the clam-shell-like opening and closing of the EAL dimer appears to be a central regulatory mechanism that seems governed by the environment and stability of the compound helix positioned at the center of this transition. Recently, an unusual quaternary arrangement was observed for RocR that resembles the in-solution conformation [40] of this REC-regulated EAL protein and reveals an intertwined tetrameric assembly that combines two EAL dimers with one EAL active site each blocked by a REC domain. In contrast, the RocR EAL domains that appear competent for catalysis seem to retain a similar regulation mechanism with sensory modules in close proximity to the compound helices and elements involved in this interaction featuring changes in deuterium incorporation upon activation of the REC domains [11]. This central role of the EAL compound helix is further supported by our HDX experiments and mutational studies on BlrP1 and by similar experimental approaches for RocR where this region was identified as a key regulatory element [11]. In terms of controlling PDE activity, this makes sense due to the direct connection of the compound helix and both β5_E_ and β6_E_ that contain conserved residues involved in metal coordination (Asp302 and Asp303) and water activation (Lys323). The reduced activity observed for protein variants with amino acid substitutions in the compound helix is likely due to altered metal coordination, which is indicated by the significant changes in their magnesium effects and calcium effects on the allosteric communication pathway as reflected in changes in their BLUF dark-state recoveries. Importantly, the compound helix of catalytically active EAL domains is highly conserved [10,11], further supporting its central role for EAL functioning even though minor modifications do not completely abolish PDE activity [13]. However, other substitutions in the compound helix can reduce activity below the detection limit as shown for BlrP1 and RocR [9,10].

Interestingly, EAL subfamilies without c-di-GMP degrading activity frequently have substitutions not only of key residues involved in catalysis but they also have degenerate compound helices [11]. While this observation supports the catalytic relevance of the compound helix due to co-evolution with key catalytic residues, it suggests that this element might have either lost its function or evolved toward a new role in these EAL families. The recently solved crystal structure of a LapD (UNP ID: Q3KK31) fragment including an EAL, a GGDEF and a signaling helix shows that, in this case, the EAL “dimerization” region has actually retained part of its function [46]. The important signaling helix that is involved in inside-out signaling of LapD binds directly to the dimerization helix and the truncated compound helix of a single EAL domain. Additional structural and biochemical data show that c-di-GMP binding to the LapD EAL domain leads to dimerization of the EAL domain and prevents the association with the signaling helix due to structural changes induced in compound helix residues [46]. Importantly, the dimeric LapD EAL assembly in the presence of c-di-GMP resembles the arrangement of catalytically active EAL domains shown above. This example nicely illustrates how allosteric changes induced by binding of the second messenger to inactive EAL domains can be used for the regulation of cellular processes employing mechanisms that were originally important for regulating PDE activity. Another degenerate EAL system that is evolutionary related to BlrP1 is YcgF from *Escherichia coli* (UNP ID: P75990). In this case, a BLUF sensor is connected to an EAL domain that has lost both its c-di-GMP binding and its c-di-GMP degrading activity but gained the function as an anti-repressor [15]. This new role is mediated via direct protein–protein interaction with a transcriptional repressor and enables a blue-light regulation of processes that are indirectly involved in c-di-GMP associated processes such as biofilm formation [47]. Considering the evolutionary relationship with BlrP1, it is suggestive that the light signal is still transmitted to the EAL dimerization region modulating the affinity of the interaction partner that might bind in this region similar to the situation in LapD.

In line with the important role of c-di-GMP in bacterial signaling, different protein–protein interactions involved in various biological processes have evolved. For many degenerate EAL systems, molecular details of their interaction interface are not known. However, recently, a study of the *Xanthomonas campestris* FimX (UNP ID: Q8P8F1) protein revealed a unique interaction of its degenerate EAL domain with a noncanonical type II PilZ domain mediated by the EAL-coordinated c-di-GMP [48]. Thus, c-di-GMP binding can directly control protein–protein interaction, but its interaction with the FimX homologue from *Pseudomonas aeruginosa* (UNP ID: Q9HUK6) was shown to also induce long-range allosteric modulations of distant REC domain [17]. In this case, both the REC domain and the EAL domain might be involved in homo- and hetero-oligomerization, which ultimately affects localization of FimX to a single pole for correct pilus assembly [16,17]. Importantly, HDX analysis of c-di-GMP binding showed also an effect on the compound helix element in this case [17], and considering the central role of this region in the previously discussed examples, it might also be involved in propagating the long-range structural changes in *P*. *aeruginosa* FimX.

In conclusion, we have shown that regulation of EAL activity in BlrP1 is centrally coordinated by the EAL dimerization interface. The compound helix plays a crucial role for the correct assembly of the EAL dimer with consequences for metal coordination in the active site and hence PDE activity. Positioning of sensory modules close to this dimerization region enables the integration of different signals into c-di-GMP metabolism, which, due to the importance of this bacterial second messenger, is reflected in various environmental stimuli-controlled EAL domains. Common features of the corresponding signaling pathways appear to be conserved even in non-catalytic EAL domains. Thus, we provide insight into the functioning of EAL domains that appears to be directly related to their central position in regulation of various cellular processes mediated by the bacterial second messenger c-di-GMP.

## Materials and Methods

### Protein expression and purification

Two single point mutations in BlrP1 were introduced by site-directed mutagenesis using PCR amplification of the pET_MBP_BlrP1 vector described in Ref. [10] according to the QuikChange protocol (Stratagene) with the following primer pairs. All sequences are shown in 5′–3′ orientation and the mutated codon is underlined: BlrP1 Y308F, forward GACTTTGGCGCAGGTTTCTCCGGCCTGTCGTTA and reverse TAACGACAGGCCGGAGAAACCTGCGCCAAAGTC; BlrP1 R316M, forward CTGTCGTTACTGACCATGTTTCAGCCTGATAAAATC and reverse GATTTTATCAGGCTGAAACATGGTCAGTAACGACAG. Verification of successful mutagenesis was performed by DNA sequencing.

Expression and purification of wild-type BlrP1 and of the two variants followed the published protocol for full-length BlrP1 [10], while the BlrP1 BLUF domain was isolated according to Ref. [36]. All proteins were prepared under safe-light conditions and finally concentrated in storage buffer [25 mM Tris–Cl, 40 mM NaCl, 5 mM MgCl_2_, 2 mM EDTA, 2 mM dithioerythritol and 5% (w/v) glycerol]. Small aliquots were flash frozen in liquid nitrogen and subsequently stored at − 80 °C.

### EAL activity assays

PDE activity was assayed as described previously [10]. This includes the bovine serum albumin pretreatment of reaction vessels, the conditions for illumination, the quenching step and the HPLC analysis. Standard conditions of this assay refer to 1.1 μM protein, 100 μM c-di-GMP, 50 mM Tris–HCl (pH 7.5), 50 mM NaCl and 10 mM MgCl_2_ at 20 °C.

### UV–Vis spectroscopy

Photocycle properties of BlrP1 and the isolated BlrP1 BLUF domain were analyzed with an experimental setup designed to minimize spectral artifacts originating from the measuring light. To this end, we used a balanced deuterium tungsten lamp (DH-2000-BAL; Mikropack), which was intensity reduced with a neutral density OD10 filter, as measuring light source and a royal blue light-emitting diode (LED) (λ_max_ = 455 nm; Doric Lenses) as excitation light source. The sample was kept at 10 °C throughout the time course of the experiment using a Peltier controlled cuvette holder connected to a TC 125 temperature controller (Quantum Northwest). Blue-light illumination was performed for 10 s with an intensity of 0.6 mW cm^− 2^ at 450 nm at the sample position. Data were continuously acquired in “kinetic” mode for 15 min employing a spectrograph coupled to an electron multiplying charge-coupled device detector (Andor Technology). Recorded spectra were averaged from 6 accumulations of 0.15 s integration time each. The change in absorbance at 502 nm was fitted with a single exponential decay function. For addressing different metal and substrate states, we diluted the proteins to concentrations between 50 and 100 μM in the appropriate buffer systems that were all set to pH 8 at 10 °C accounting for their temperature dependence. Dilutions for the photocycle experiments in the Mg^2 +^-bound state were performed using buffer A [25 mM Tris–Cl, 40 mM NaCl, 5 mM MgCl_2_, 2 mM EDTA and 5% (w/v) glycerol]. The effect of Mn^2 +^ was tested in buffer B [25 mM Tris–Cl, 40 mM NaCl, 10 mM MnCl_2_, 2 mM EDTA and 5% (w/v) glycerol], and dilutions for Ca^2 +^ were prepared with buffer C [25 mM Tris–Cl, 40 mM NaCl, 30 mM CaCl_2_, 2 mM EDTA and 5% (w/v) glycerol]. For removing the metal ions from the active site, we diluted in Mg^2 +^-free buffer A and incubated the sample in the presence of 20 mM EDTA for 15 min prior to data acquisition. The effect of c-di-GMP binding was tested by including the substrate at a final concentration of 47 μM in either the Ca^2 +^-treated sample or the EDTA-treated sample of 50 μM wild-type BlrP1.

### Hydrogen–deuterium exchange–mass spectrometry

All samples for HDX were prepared under safe-light conditions except where noted otherwise. For addressing light- and/or substrate-induced structural changes, we performed four labeling experiments: BlrP1 in the presence of Mg^2 +^ (Mg) or inhibiting concentrations of Ca^2 +^ and c-di-GMP present (Cc) under dark (d) or blue-light (l) conditions. The corresponding experimental setups are referred to as Mg_d_, Mg_l_, Cc_d_ and Cc_l_, respectively, in the main text. To this end, we pre-incubated BlrP1 (108 μM) in buffer A at 10 °C for 1 min in the dark or with parallel illumination from a royal blue (455 nm) collimated LED lamp (Thorlabs) providing a light intensity of 1 mW cm^− 2^ at the sample position (Mg_d_ or Mg_l_, respectively). Ca^2 +^-inhibited samples were prepared by pre-incubation of BlrP1 at 4 °C in buffer C for 5 min followed by addition of c-di-GMP and another 5 min incubation step. Final concentrations were 108 μM BlrP1 and 611 μM c-di-GMP and 30 mM Ca^2 +^. Equilibration of the samples for either dark-state or light-state measurements (Cc_d_ or Cc_l_, respectively) was performed as described above for Mg_d_ or Mg_l_, respectively. The corresponding light conditions were maintained throughout the labeling procedure. For this purpose, 2-μL aliquots of the equilibrated samples were diluted with 38 μL buffer A_D_ or buffer C_D_ [corresponding to buffers A and C, however, prepared with D_2_O and glycer(*d*_3_-ol) including the temperature and D_2_O corrections for pD 8.0 at 10 °C] and 5-μL samples were removed after 15 s, 1 min, 5 min, 20 min and 60 min. Deuterium incorporation was terminated by quenching the samples in 55 μL of 200 mM ice-cold NH_4_ formic acid (pH 2.6) of which 50 μL was then injected into a cooled HPLC setup.

Deuterated samples were digested on a pepsin column (Applied Biosystems) kept at 10 °C. All subsequent chromatographic steps were carried out in a water bath at 0.5 ± 0.1 °C. Peptic fragments were buffer-exchanged on a 2-cm C_18_ guard column (Discovery Bio C_18_ packing) and separated in the presence of 0.6% formic acid with a 15-min acetonitrile gradient (15–50%) on a reversed phase column (Discovery Bio Wide Pore C_18_ 10 cm × 1 mm − 3 μm; Supelco). Eluting peptides were infused into a maXis electrospray ionization–ultra high resolution–time-of-flight mass spectrometer (Bruker) for measuring the extent of deuterium incorporation. We analyzed deuterium incorporation with an improved version of the automated software package Hexicon [49]. This in-house version (manuscript in preparation) is based on the previously published NITPICK algorithm for feature detection [50] and Hexicon for the analysis of deuterium incorporation. Deuterium incorporation was quantified in triplicate measurements by the mean shift of a peptide's mass centroid. Deuterium incorporation plots provided in Fig. 2 and Figs. S2–S6 show the mean relative deuterium incorporation at each exchange time point with error bars reflecting the standard deviation of triplicate data points. The absolute difference of a peptide's relative deuterium incorporation (Δ*D*_rel_) between two states (i.e., light adapted *versus* dark adapted) was used to evaluate the changes in structural dynamics of the corresponding protein region. Assignment of colors according to the legends in Figs. 3 and 4 accounts for the calculated standard deviation, and therefore, only statistically significant changes in deuterium incorporation are highlighted. As an additional quality control of data processing, estimated abundance distributions are provided in the sub-panels of Fig. 2 and Figs. S2–S6. Briefly, this deuterium incorporation distribution corresponds to a deconvolution of the average extracted isotope pattern taking into account the natural isotope distribution. Therefore, it provides an estimate of the abundance of species with a given number of deuterons incorporated that are plotted on a scale from undeuterated (0) to all exchangeable amides deuterated [number of amino acids except prolines minus 1 (due to rapid back-exchange of the N-terminal amine group)].

### SAXS and NMA

SAXS data were collected at the X12SA cSAXS beamline at the Swiss Light Source (Villigen, Switzerland). A series of protein concentrations between 2 and 20 mg mL^− 1^ were measured in buffer containing 25 mM Hepes, 100 mM NaCl, 5 mM MgCl_2_, 2.5 mM EDTA, 2 mM dithioerythritol and 10% glycerol at pH 7.5. The samples were mounted in Ø 1-mm-quartz capillaries under dimmed red light and kept at 11 °C throughout the experiment. For light activation of the BLUF domain, two LEDs (λ_max_ = 455 nm; Doric Lenses) were mounted on two sides of the capillary and each focused with a cylindrical lens to provide a light power of 10 mW cm^− 2^ each. Data acquisition using 12.4 keV photons and an X-ray beam diameter of 300 μm was performed in 330 μm steps along the capillary with 4 × 0.25 s exposures at each position. Scattered X-rays were recorded with a Pilatus 2M detector positioned at a distance of 2.2 m from the sample. Data were collected from the buffer alone and from the protein in the dark and under constant illumination with both LEDs. The order of dark- and light-state data acquisition was varied to check for the effect of radiation damage and the reversibility of structural changes upon light activation. All data were azimuthally integrated and averaged. The scattering vector *q* is defined as *q* = 4πsin(θ)λ^− 1^.

For data analysis, the buffer signal was subtracted from that of the buffered protein solution. Guinier plots of the lowest protein concentration data are shown in Fig. S8a. To reduce possible aggregation artifacts in the low *q*-region of high concentration samples, data from the lowest concentration sample were merged with data from the highest concentration at *q* = 0.09 Å^− 1^ for both the dark state and the light state. Kratky plots corresponding to the globular two domain architecture of BlrP1 are shown in Fig. S8b. CRYSOL 2.6 [51] with default parameters was used to fit the crystal structure and the structures obtained by NMA with the NOMAD-Ref Web server [52] to the scattering data. The initial input model for the analysis of normal modes was the published crystal structure of BlrP1 PDB ID: 3GG0 [10]. Out of the 30 generated substructures that correspond to one full cycle of the normal mode transition, structures #8 and #23 correspond to the maximal amplitude of structural changes. As structure #23 of normal mode 8 best explained the dark-state data, it was taken as a starting structure for an additional NOMAD-Ref analysis to compare the results with light-induced structural rearrangements observed by SAXS.

The following are the supplementary data related to this article.

Supplementary data and supplementary figures.

Movie S1 Animation of time-dependent changes in deuterium incorporation upon substrate and calcium binding in the dark. The time series of Cc_d_–Mg_d_ comparisons is presented with colors corresponding to the differences in *D*_rel_ according to the bar legend. Red or blue colors reflect an increased or decreased deuterium uptake, respectively, upon substrate coordination and calcium coordination. FMN and c-di-GMP are shown as yellow and orange stick models, respectively, and metal centers as purple spheres. Individual structural elements correspond to details of Fig. 3a.

Movie S2 Animation of time-dependent changes in deuterium incorporation upon illumination in the presence of substrate and calcium. The time series of Cc_l_–Cc_d_ comparisons is presented with colors corresponding to the differences in *D*_rel_ according to the bar legend. Red or blue colors reflect an increased or decreased deuterium uptake, respectively, upon illumination in the presence of c-di-GMP and Ca^2 +^. FMN and c-di-GMP are shown as yellow and orange stick models, respectively, and metal centers as purple spheres. Individual structural elements correspond to details of Fig. 3b

Movie S3 Animation of time-dependent changes in deuterium incorporation upon illumination in the absence of substrate and with Mg^2 +^ present. The time series of Mg_l_–Mg_d_ comparisons is presented with colors corresponding to the differences in *D*_rel_ according to the bar legend. Red or blue colors reflect an increased or decreased deuterium uptake, respectively, upon illumination of BlrP1 in the presence of Mg^2 +^. FMN and c-di-GMP are shown as yellow and orange stick models, respectively, and metal centers as purple spheres. Individual structural elements correspond to details of Fig. 3c

Movie S4 Animation of time-dependent changes in deuterium incorporation upon substrate and calcium binding in light-adapted BlrP1. The time series of Cc_l_–Mg_l_ comparisons is presented with colors corresponding to the differences in *D*_rel_ according to the bar legend. Red or blue colors reflect an increased or decreased deuterium uptake, respectively, upon substrate coordination and calcium coordination in the light-adapted BlrP1 state. FMN and c-di-GMP are shown as yellow and orange stick models, respectively, and metal centers as purple spheres. Individual structural elements correspond to details of Fig. 3d.

Movie S5 Animation of the normal mode best describing the differences between experimental SAXS data and the crystal structure. Thirty substructures of normal mode 8 (NOMAD-Ref output) with the maximal amplitude between structures #8 and #23 are shown as cartoon representation. The BLUF domain is colored orange and the EAL domain is in blue. Flavin and c-di-GMP are omitted for clarity.

Movie S6 Animation of the normal mode representing the light-induced structural changes observed for BlrP1. Thirty substructures corresponding to one full cycle of normal mode 10 (NOMAD-Ref output) are shown as cartoon representation. The BLUF domain is colored orange and the EAL domain is in blue. Flavin and c-di-GMP are omitted for clarity.

*Klebsiella pneumoniae* BlrP1 pH 9.0 manganese/c-di-GMP complex.

Supplementary data to this article can be found online at http://dx.doi.org/10.1016/j.jmb.2013.11.018.

## Figures and Tables

**Table 1:** Dark-state recovery of BlrP1 and its isolated BLUF domain in the presence or absence of various divalent metals and the substrate c-di-GMP at 10 °C.

Experiment	Mean lifetime, τ (s)
BlrP1–Mg^2+^	326 ± 4
BlrP1–Mn^2+^	351 ± 8
BlrP1–Ca^2+^	208 ± 5
BlrP1–Ca^2+^–c-di-GMP	182 ± 7
BlrP1–EDTA	258 ± 4
BlrP1–EDTA–c-di-GMP	230 ± 5
BlrP1 Y308F–Mg^2+^	264 ± 4
BlrP1 Y308F–Ca^2+^	177 ± 3
BlrP1 Y308F–EDTA	252 ± 6
BlrP1 R316M–Mg^2+^	281 ± 7
BlrP1 R316M–Ca^2+^	185 ± 5
BlrP1 R316M–EDTA	259 ± 6
BlrP1 BLUF–Mg^2+^	212 ± 4
BlrP1 BLUF–Ca^2+^	210 ± 4
BlrP1 BLUF–EDTA	226 ± 4

Mean lifetimes are stated as the mean of four repetitive light–dark cycle measurements ± standard deviation.

**Table 2:** Enzymatic activity of c-di-GMP hydrolysis to 5′-pGpG for BlrP1 and two variants measured under standard conditions as described in Ref. [10].

Protein	*k*_cat_ (s^− 1^) (dark)	*k*_cat_ (s^− 1^) (light)
BlrP1 wild type [10]	0.13 ± 0.02	0.54 ± 0.02
BlrP1 Y308F	0.014 ± 0.007	0.06 ± 0.03
BlrP1 R316M	0.029 ± 0.007	0.10 ± 0.06
