# Supplementary material for: Characterization of Elements Involved in Allosteric Light Regulation of Phosphodiesterase Activity by Comparison of Different Functional BlrP1 States
Source: J Mol Biol. 2014 Feb 20;426(4):853–68. doi: 10.1016/j.jmb.2013.11.018 (PMC3989770; doi:10.1016/j.jmb.2013.11.018)
Supplement: Supplementary file 1 — Supplementary data and supplementary figures. [file mmc1.pdf]

Supplementary Information

for

Characterization of elements involved in allosteric light-regulation of phosphodiesterase activity by comparison of different functional BlrP1 states

Andreas Winkler[1,*], Anikó Udvarhelyi[1], Elisabeth Hartmann[1], Jochen Reinstein[1], Andreas Menzel[2], Robert L. Shoeman[1] and Ilme Schlichting[1,*]

[1] Department of Biomolecular Mechanisms, Max Planck Institute for Medical Research, Heidelberg, 69120, Germany

[2] Coherent X-ray Scattering Group, Paul Scherrer Institute, Villigen, 5232, Switzerland

Correspondence should be addressed to A.W. (Andreas.Winkler@mpimf-heidelberg.mpg.de) or I.S. (Ilme.Schlichting@mpimf-heidelberg.mpg.de)

Supplementary SAXS results

In order to better understand global structural rearrangements upon illumination, we combined small-angle X-ray scattering (SAXS) studies of BlrP1 under conditions resembling $\mathbf{Mg_d}$ and $\mathbf{Mg_l}$ with normal mode analysis (NMA). The CRYSOL-fit of the crystal structure in complex with manganese and c-di-GMP (PDB ID: **3GG0**[10]) showed a good overall correspondence with the measured dark state ($\mathbf{Mg_d}$) radial density distribution of BlrP1 and their comparison revealed clear discrepancies only between q = 0.12-0.22 $Å^{-1}$ (Fig. S7a). This suggests a slightly different conformation in solution with a possibly different arrangement of the domains within the dimeric BlrP1 assembly. This may be related to the differences in experimental conditions of our SAXS measurements and crystallization setups, i.e. nucleotide free BlrP1 in the presence of Mg^{2+} versus the Ca^{2+}-c-di-GMP complex, respectively. While the variation between individual *ab initio* reconstruction of protein structures using dummy atom representations[53] did not allow a meaningful interpretation of the structural differences, NMA using the NOMAD-Ref server[52] proved useful to rationalize the differences in the scattering curves. One specific normal mode (mode 8), which corresponds to an opening-closing of the EAL dimer, explained the discrepancy between the initial fit of the crystal structure and the solution scattering data. A CRYSOL-fit of one substructure of this normal mode (substructure #23, Fig. S7a-b), which represents a more closed EAL dimer arrangement than in the crystal structure, reproduced the experimental data (Fig. S7a-c, Movie S5). Interestingly, the conformations of the EAL domains during the clam-shell opening of BlrP1 reflect the characteristic difference in EAL dimer arrangements observed in various EAL structures deposited in the PDB (Fig. 5), which further supports the functional relevance of the opening-closing movement of the EAL dimer. While changes in the EAL dimer interface could correspond to experimental conditions of substrate-binding or metal-coordination, no clear trend can be observed when comparing the structures shown in Figure 5[10; 13; 24; 39; 40].

Interestingly, the characteristic compound helix element is disordered in the most open EAL dimer conformation in the absence of metals and substrate[39].

Importantly, the comparison of SAXS data obtained under conditions resembling $\mathbf{Mg_d}$ and $\mathbf{Mg_l}$ also indicated an inter-domain movement due to blue-light illumination of BlrP1. The light-dark difference solution scattering trace showed a distinct and reproducible signal between q = 0.1-0.2 $Å^{-1}$ with a minimum at q = 0.14 $Å^{-1}$, suggesting inter-domain rearrangements upon blue-light illumination (Fig. S7d). Again, NMA allowed the identification of a specific mode that reproduced the light-dark scattering curve (mode 10, Fig. S7d-e). CRYSOL-fits of a specific substructure of normal mode 10 (substructure #4) matched the experimental solution scattering data well (Fig. S7d-f, Movie S6) and the difference in radial density distributions with substructure #23 of normal mode 8, representing the dark state, correctly reproduced the measured SAXS light-dark difference signal (Fig. S7d inset). Fits with other substructures of any normal mode neither explained the light-dark difference data satisfactorily nor did they reproduce the minimum at q = 0.14 $Å^{-1}$. The structural movement of normal mode 10 corresponds to a twisting mode of both the EAL and BLUF domains, which is accompanied by a subtle opening-closing motion of the EAL dimer. While individual *ab initio* reconstructions showed a variability that prevented a qualitative interpretation, similar structural rearrangements were also suggested by 3D reconstructions of the experimental data using BUNCH[54]. However, they need to be considered with care (Fig. S8c) because BUNCH, in contrast to CRYSOL**,** does not take into account the hydration shell. Therefore the CRYSOL-computed scattering curves of the resulting BUNCH structures significantly deviated from the data. This prevented its use to model our measured difference signal and, hence, we exclusively compared fits obtained with CRYSOL.

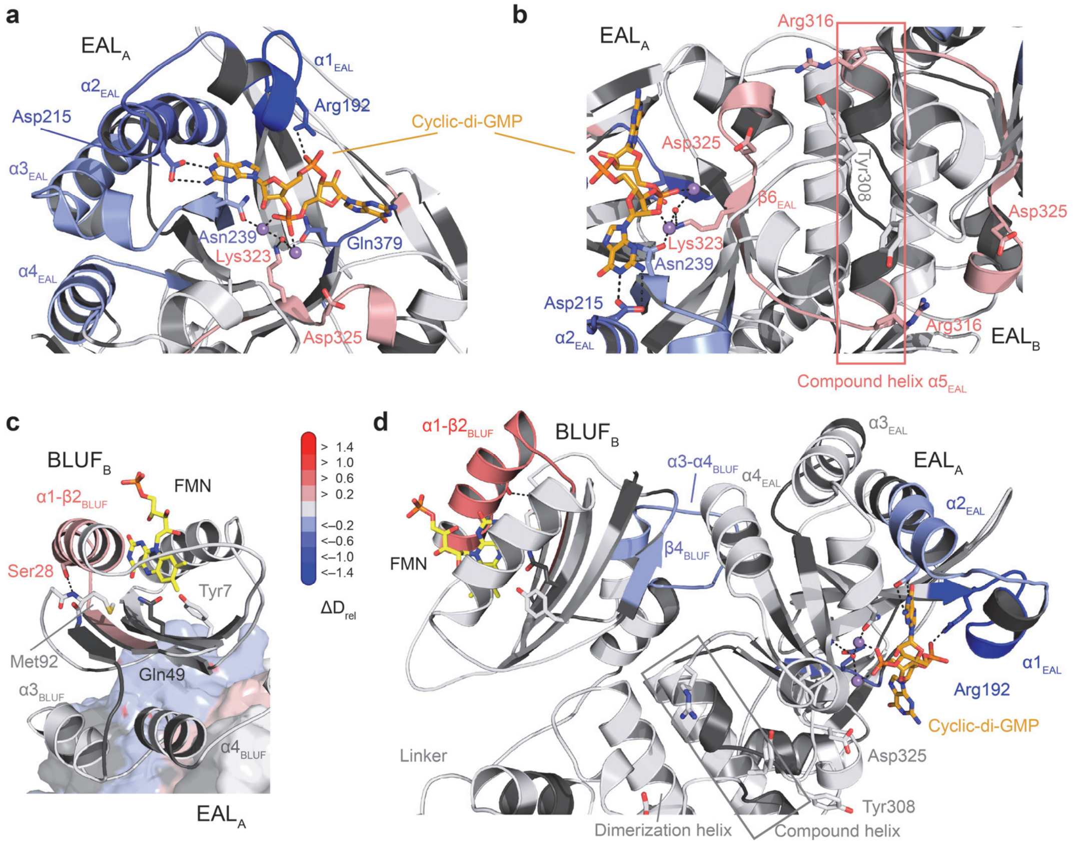

Figure S1. Close-up of functionally important BlrP1 regions identified by HDX. Colors of structural elements correspond to a representative difference in relative deuterium incorporation according to the scale located in the middle of the figure. For panels a-c the 5 minute exchange time point of the $\mathbf{Cc_d}$ – $\mathbf{Mg_d}$ comparison is shown and panel d corresponds to the 1 minute deuteration time point of the $\mathbf{Cc_l}$ – $\mathbf{Mg_l}$ comparison. FMN and c-di-GMP are shown as yellow and orange stick models, respectively. (a) Active site of one EAL protomer (chain A) with functionally important residues shown as stick models. (b) Close-up of the EAL dimerisation region highlighting the central positioning of the compound helix and its direct connection to the active site. (c) Details of the BlrP1 BLUF domain (chain B) showing the central Tyr7-Gln49-Met92 triad and the hydrogen bond between the Met92 amide and the Ser28 side chain. The EAL domain is shown in surface mode. (d) Overview of structural elements positioned at the evolutionarily conserved BLUF-EAL interface.

Figures S2-S5. Details of all evaluated peptides from HDX analysis of BlrP1. Four full page views of the individual comparisons from Figure 3a-d are provided with deuterium incorporation plots of all 90 peptides. Please zoom in on the region of interest for full details. Figures S2, S3, S4 and S5 correspond to $\mathbf{Cc_d – Mg_d}$, $\mathbf{Cc_l – Cc_d}$, $\mathbf{Mg_l – Mg_d}$ and $\mathbf{Cc_l – Mg_l}$, respectively. Individual deuterium incorporation plots show the time dependent increase in relative deuterium levels in the main panel. D_{rel} values are shown as the mean of three independent measurements and error bars correspond to the standard deviation. A software-estimated abundance distribution of deuterated species is presented in the lower sub-panel on a scale from undeuterated to all exchangeable amides deuterated. Importantly, we do not observe any isotope distribution that indicates the correlated exchange regime (EX1).

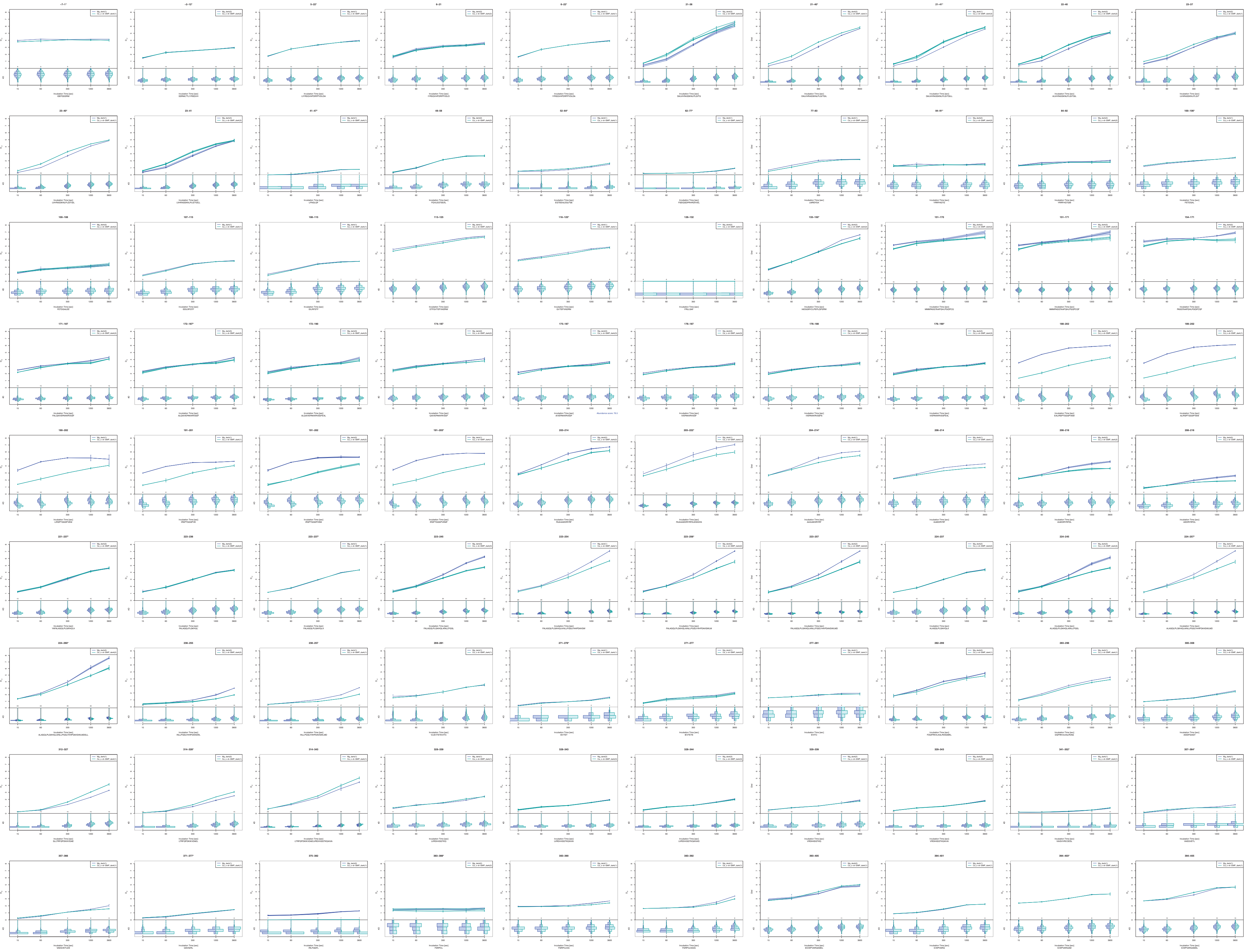

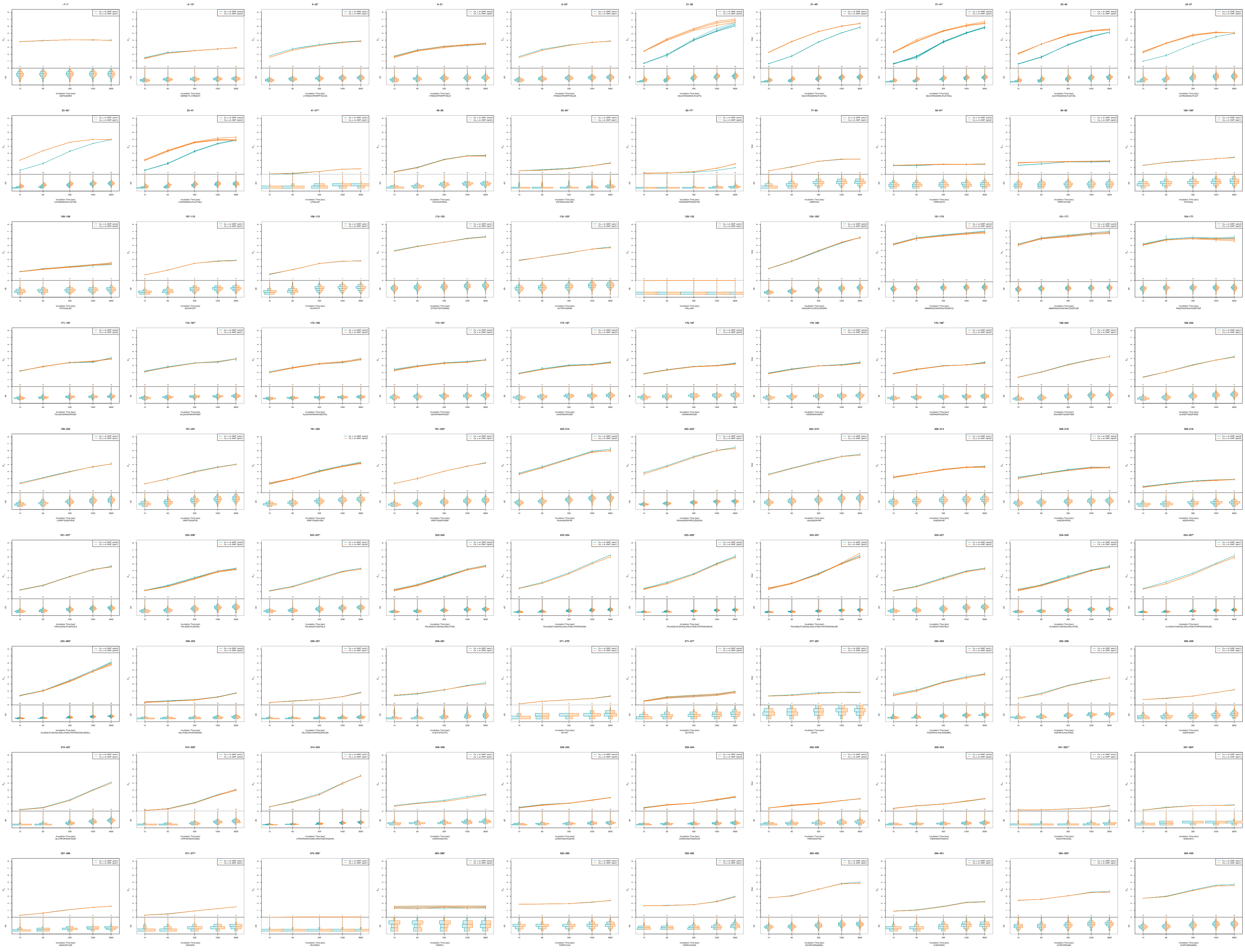

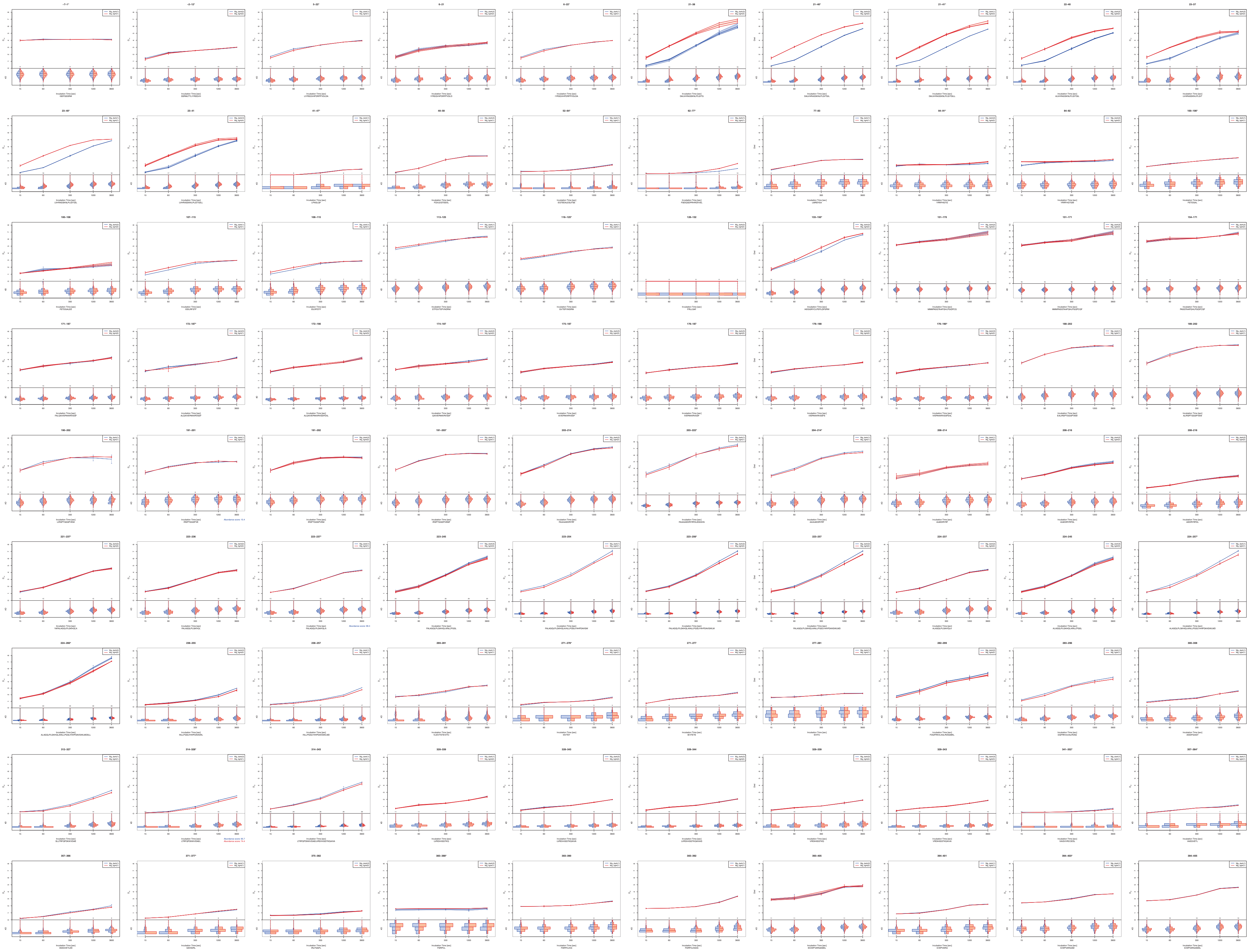

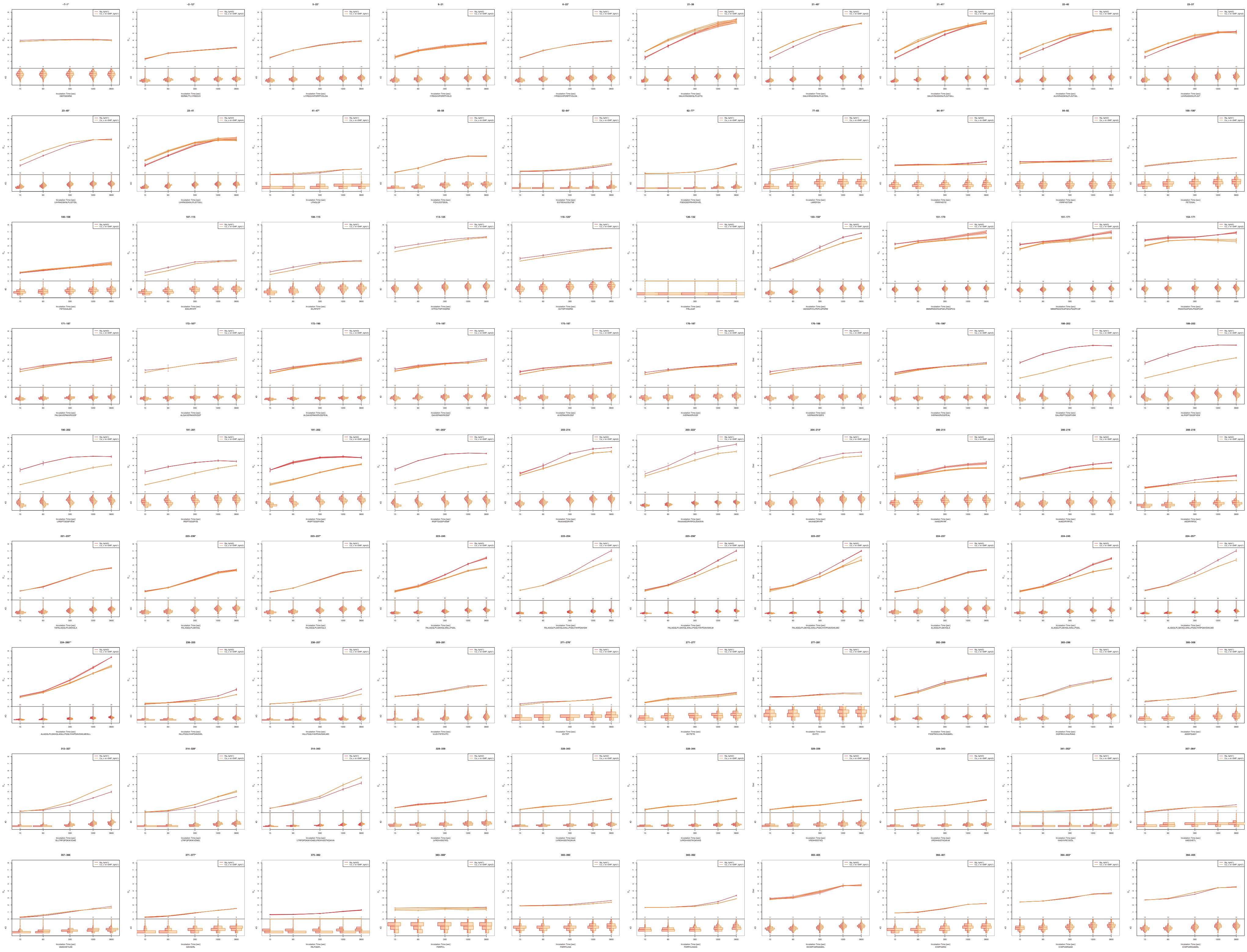

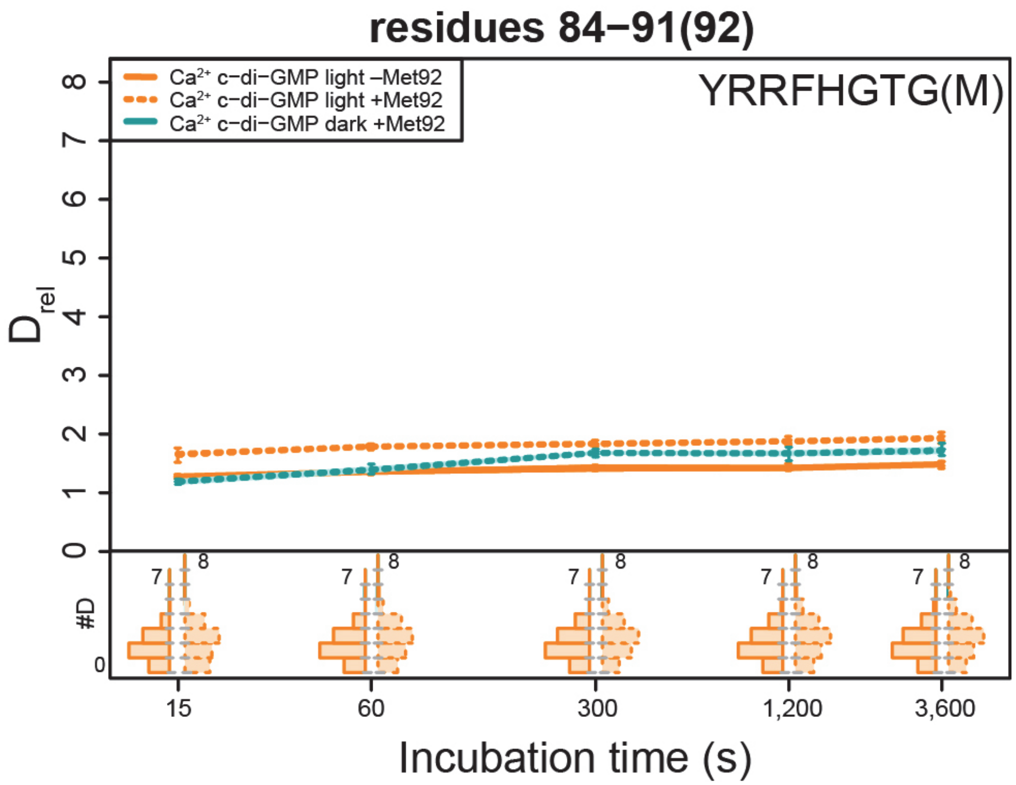

Figure S6. Deuterium incorporation plot of two overlapping β5$_B$ BlrP1 peptides at different experimental conditions. Labeling-time dependence of relative deuterium incorporation is shown for **Cc$_d$** and **Cc$_l$** in turquoise and orange, respectively. The estimated abundance distribution of individual deuterated species is presented in the lower sub-panel only for the overlapping peptides in **Cc$_l$** on a scale from undeuterated to all exchangeable amides deuterated. The straight line belongs to the peptide including residues 84-91 and dashed lines correspond to region 84-92. D_{rel} is not corrected for back-exchange during the HDX experiments and hence the observed ΔD_{rel} of ~0.6 corresponds to a difference in one amide bond based on the back-exchange of 38 % estimated using fully exchanging peptides from flexible regions. D_{rel} values are shown as the mean of three independent measurements and error bars correspond to the standard deviation.

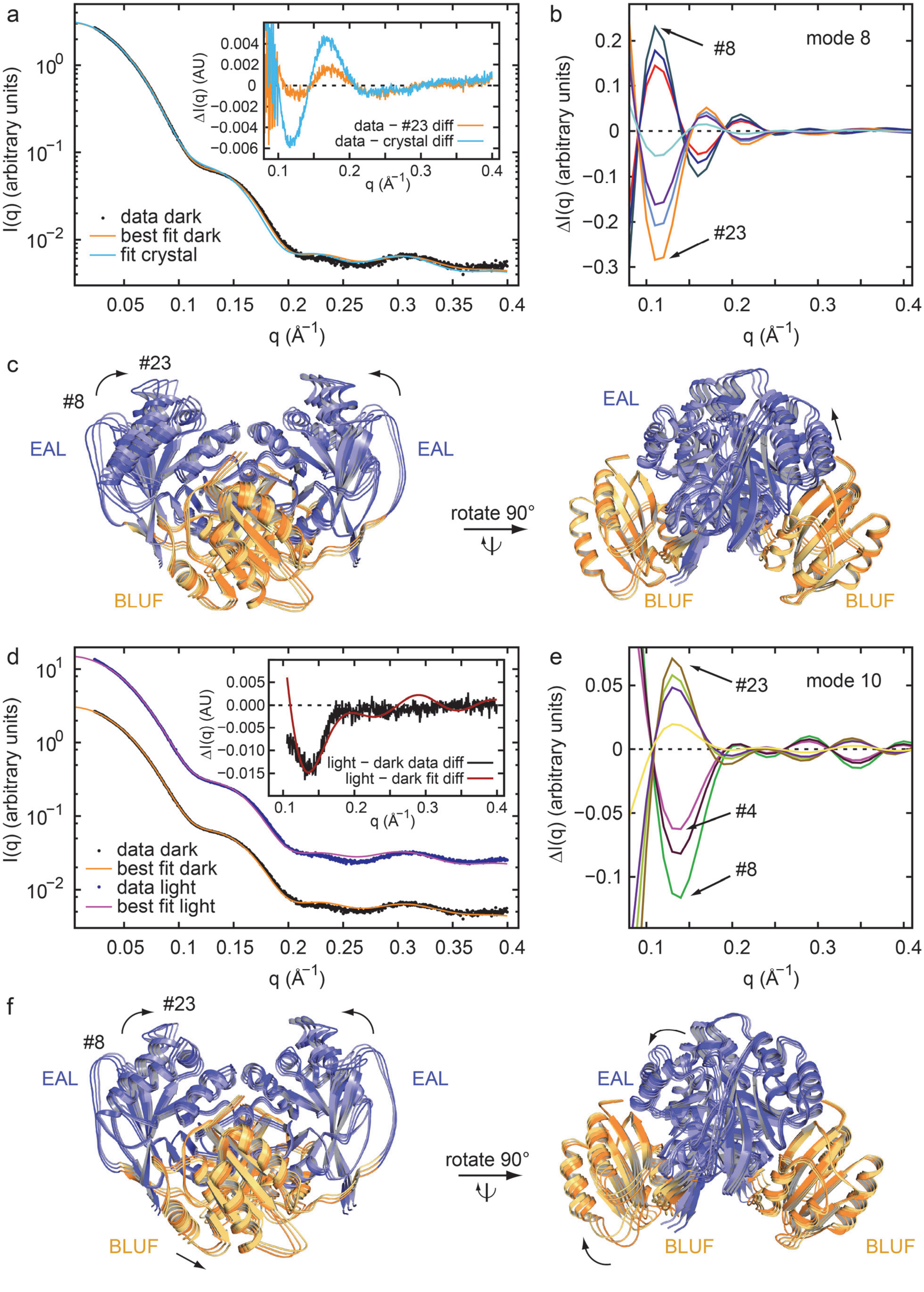

a
I(q) (arbitrary units)
q (Å^{-1})
data dark
best fit dark
fit crystal
ΔI(q) (AU)
data − #23 diff
data − crystal diff
b
mode 8
#8
#23
ΔI(q) (arbitrary units)
c
#23
#8
EAL
EAL
BLUF
rotate 90°
EAL
BLUF
BLUF
d
data dark
best fit dark
data light
best fit light
light − dark data diff
light − dark fit diff
e
mode 10
#23
#4
#8
f
#23
#8
EAL
EAL
BLUF
rotate 90°
EAL
BLUF
BLUF

Figure S7. SAXS data and normal mode analysis. (a) Scattering curve of dark-adapted BlrP1 fitted with substructure #23 of normal mode 8 (orange) and with the crystal structure (cyan) using CRYSOL. Inset – Difference radial density distribution of the measured data with the CRYSOL-calculated scattering curves of substructure #23 of normal mode 8 and the crystal structure. (b) Differences of CRYSOL-calculated scattering curves of selected substructures of normal mode 8 with the scattering curve of the crystal structure. Substructure #23 of normal mode 8 with a more closed EAL conformation describes the solution data better than the crystal structure. (c) Representative structures for the maximal amplitude of normal mode 8 are shown as cartoon representation. The BLUF domain is colored in shades of orange and the EAL domain in shades of blue. Flavin and c-di-GMP are omitted for clarity. The full animation can be seen in Movie S5. (d) Solution scattering curves of dark- and light-state BlrP1. The two datasets are translated along the ordinate for better visualization. The data are fitted with substructure #23 of normal mode 8 (dark-state, orange) and with substructure #4 of normal mode 10 (light-state, pink) using CRYSOL. Inset – light-dark difference radial density distribution showing the light-induced signal around 0.14 $Å^{-1}$. For comparison, the difference of the corresponding light and dark fits is also plotted. (e) Differences of CRYSOL-calculated scattering curves of selected substructures of normal mode 10 with the scattering curve of the crystal structure. (f) Representative substructures for the maximal amplitude of normal mode 10 are shown as cartoon representation. Colors correspond to those used in panel c and the full animation of this normal mode is presented in Movie S6.

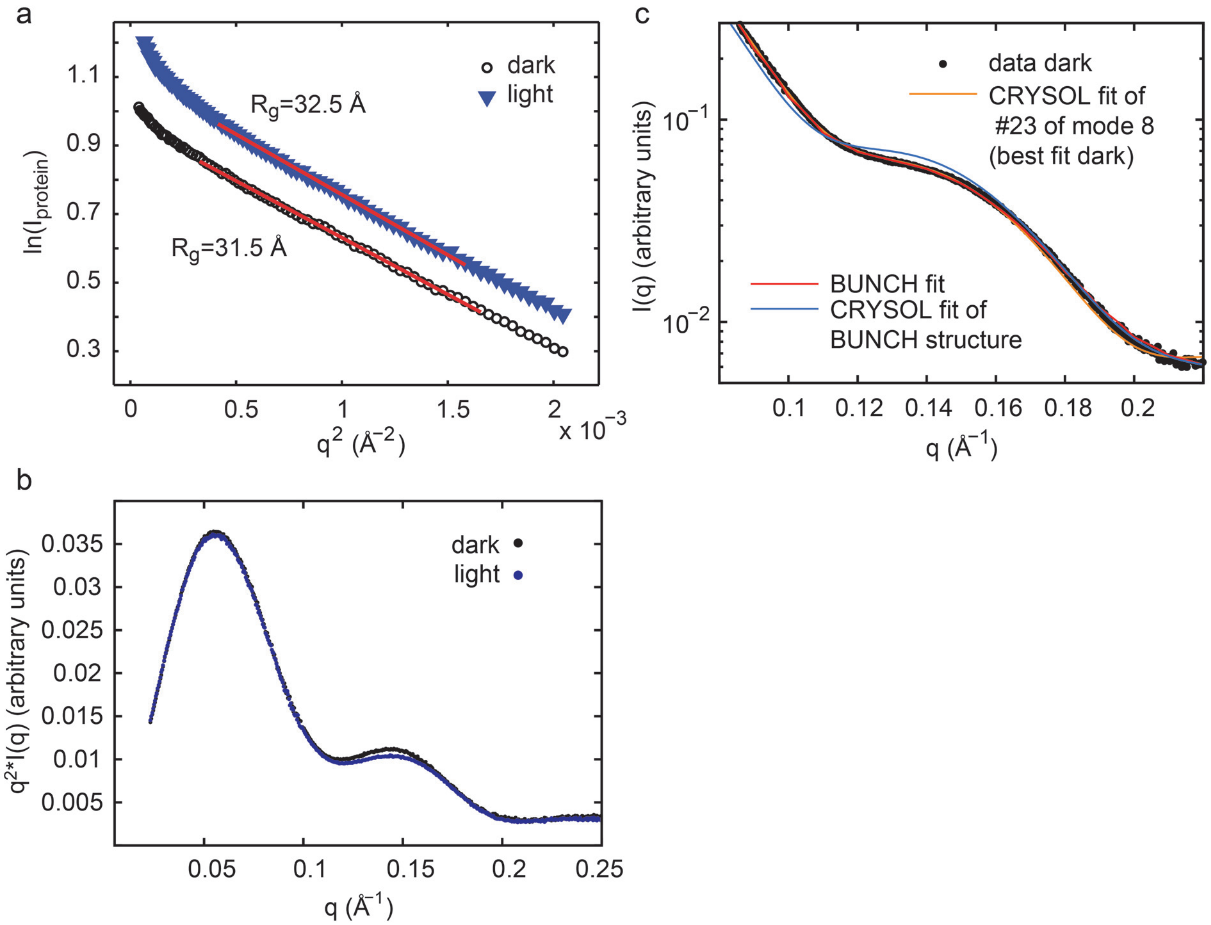

Figure S8. SAXS analysis. (a) Guinier plot of dark- and light-state data (displaced along the ordinate with arbitrary units for better visualization). The red lines indicate the regions of the fit. For light-state measurements a slight tendency for aggregation was observed. (b) Kratky plot of dark- and light-state data represented by black and blue dots, respectively. (c) Comparison of BUNCH and CRYSOL fits of a BUNCH-generated model for the dark state data in the critical q-region around 0.1-0.2$Å^{-1}$. The best CRYSOL-fit based on substructure #23 of normal mode 8 is also shown for comparison.

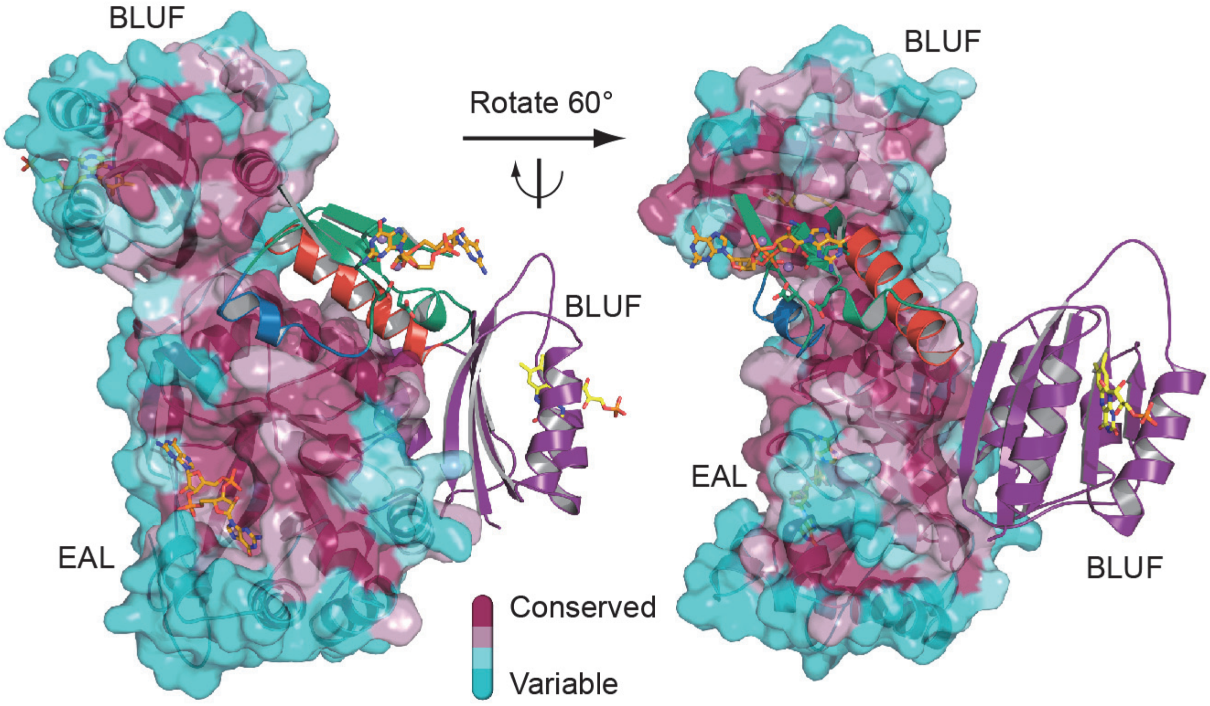

Figure S9. Evolutionary conservation of the EAL dimerization interface and the EAL contact site with the BLUF domain. A single BlrP1 protomer is shown as cartoon and transparent surface representation and colored according to the evolutionary conservation of amino acids. Conservation scores were calculated with ConSurf[55] based on a multiple alignment of 160 BLUF-EAL homologues. FMN and c-di-GMP are shown as yellow and orange stick models, respectively. Of the second BlrP1 protomer only selected regions are shown in cartoon representation with the BLUF domain in purple and parts of the EAL domain colored according to Figure 1. High conservation is observed for the EAL active site and the EAL-EAL dimerization region. In addition, residues of the compound helix environment and its contact site on the BLUF domain ($\beta 4_B$ and the $\alpha 3$-$\alpha 4_B$ loop) also show a high evolutionary conservation.

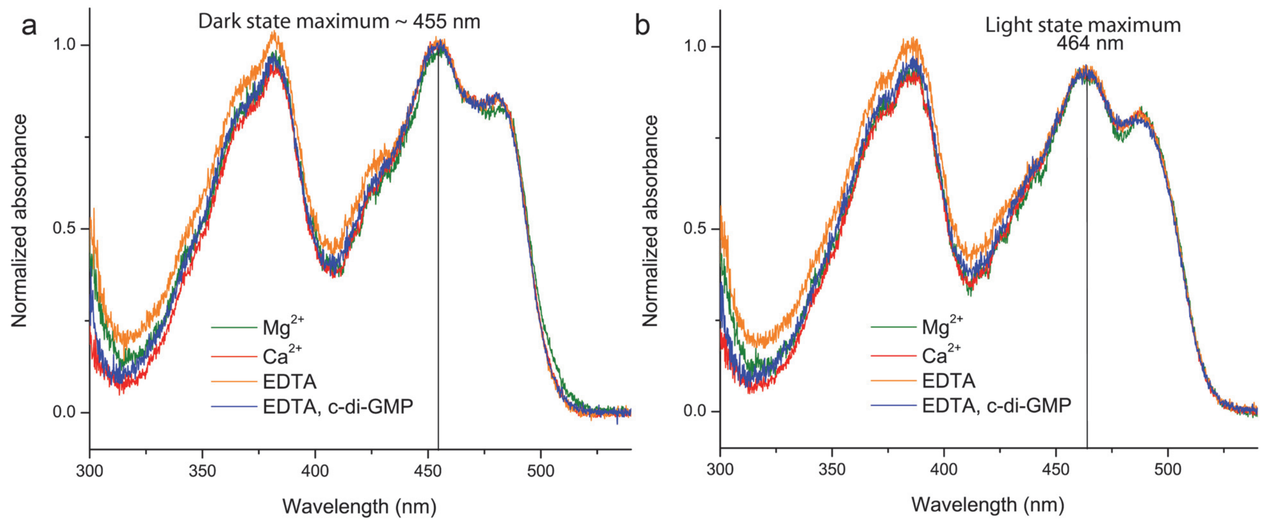

Figure S10. Absorption spectra of BlrP1 in the presence or absence of metal ions. (a) Dark state spectra of full-length BlrP1 in the presence of Mg^{2+} (green), Ca^{2+} (red), EDTA (orange) and EDTA plus c-di-GMP (blue). (b) Spectra acquired immediately after switching off the blue LED used for populating the light-activated state of BlrP1 (10 seconds with 5 mW cm^{-2} at the sample position). Color coding of individual traces corresponds to panel a. All dark state spectra were normalized corresponding to the absorption at 455 nm to enable a better comparison of individual spectra recorded at slightly different protein concentrations. Light state spectra were normalized according to the correction of their corresponding dark state spectra. Vertical black lines correspond to the maximum absorption of the S_0-S_1 flavin transition and indicate the characteristic ~10 nm red-shift of the light-activated BLUF domain. Spectra were acquired with a DU970 CCD detector (Andor Newton) and correspond to the average of six accumulations with 0.15 s integration time each.

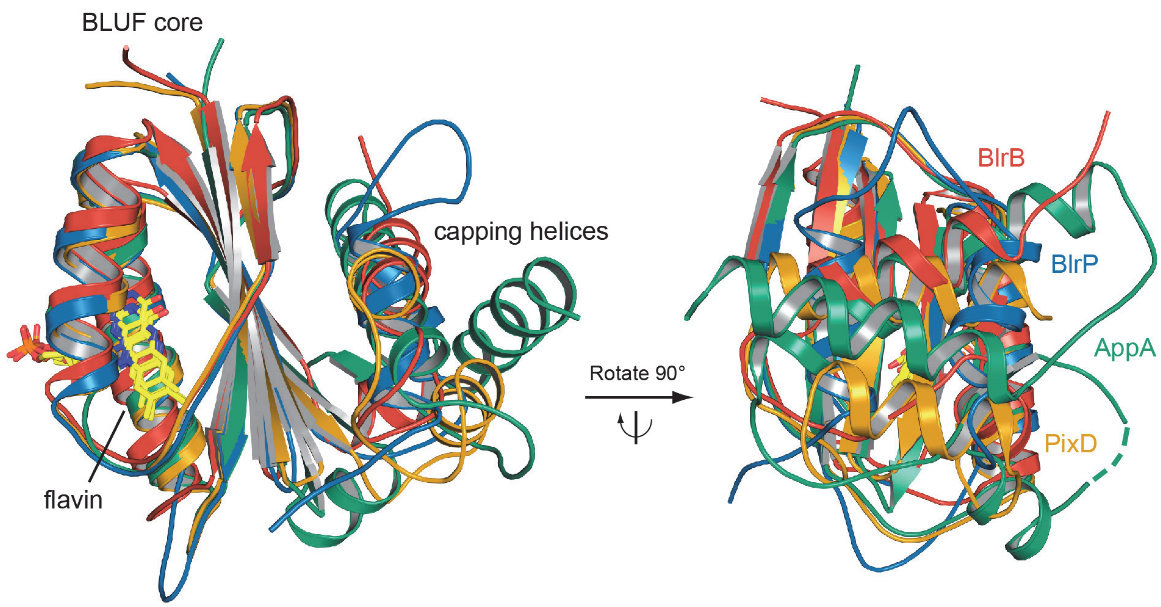

Figure S11. Structural alignment of different BLUF domains. Superposition of the BLUF domains of BlrP1– PDB ID: **3GG0**[10], AppA – PDB ID: **4HH0**[30], BlrB (UNP ID: **Q3IYE4**) – PDB ID: **2BYC**[43] and PixD (UNP ID: **P74295**) – PDB ID: **2HFN**[44] colored in blue, green, red and orange, respectively, reveals an identical fold of the BLUF core region but pronounced differences in the arrangement of the C-terminal helical extensions. With the exception of BlrP1, no other crystal structure was obtained in the presence of the corresponding effector regions.

Movie S1. Animation of time-dependent changes in deuterium incorporation upon substrate and calcium binding in the dark. The time series of $\mathbf{Cc_d}$ – $\mathbf{Mg_d}$ comparisons is presented with colors corresponding to the differences in D_{rel} according to the bar-legend. Red or blue colors reflect an increased or decreased deuterium uptake, respectively, upon substrate- and calcium-coordination. FMN and c-di-GMP are shown as yellow and orange stick models, respectively, and metal centers as purple spheres. Individual structural elements correspond to details of Figure 3a.

Movie S2. Animation of time-dependent changes in deuterium incorporation upon illumination in the presence of substrate and calcium. The time series of $\mathbf{Cc_l}$ – $\mathbf{Cc_d}$ comparisons is presented with colors corresponding to the differences in D_{rel} according to the bar-legend. Red or blue colors reflect an increased or decreased deuterium uptake, respectively, upon illumination in the presence of c-di-GMP and Ca^{2+}. FMN and c-di-GMP are shown as yellow and orange stick models, respectively, and metal centers as purple spheres. Individual structural elements correspond to details of Figure 3b

Movie S3. Animation of time-dependent changes in deuterium incorporation upon illumination in the absence of substrate and with Mg^{2+} present. The time series of $\mathbf{Mg_l}$ – $\mathbf{Mg_d}$ comparisons is presented with colors corresponding to the differences in D_{rel} according to the bar-legend. Red or blue colors reflect an increased or decreased deuterium uptake, respectively, upon illumination of BlrP1 in the presence of Mg^{2+}. FMN and c-di-GMP are shown as yellow and orange stick models, respectively, and metal centers as purple spheres Individual structural elements correspond to details of Figure 3c

Movie S4. Animation of time-dependent changes in deuterium incorporation upon substrate and calcium binding in light-adapted BlrP1. The time series of $\mathbf{Cc_l}$ – $\mathbf{Mg_l}$ comparisons is presented with colors corresponding to the differences in D_{rel} according to the bar-legend. Red or blue colors reflect an increased or decreased deuterium uptake, respectively, upon substrate- and calcium-coordination in the light-adapted BlrP1 state. FMN and c-di-GMP are shown as yellow and orange stick models, respectively, and metal centers as purple spheres. Individual structural elements correspond to details of Figure 3d.

Movie S5. Animation of the normal mode best describing the differences between experimental SAXS data and the crystal structure. 30 substructures of normal mode 8 (NOMAD-Ref output) with the maximal amplitude between structures #8 and #23 are shown as cartoon representation. The BLUF domain is colored in orange and the EAL domain in blue. Flavin and c-di-GMP are omitted for clarity.

Movie S6. Animation of the normal mode representing the light-induced structural changes observed for BlrP1. 30 substructures corresponding to one full cycle of normal mode 10 (NOMAD-Ref output) are shown as cartoon representation. The BLUF domain is colored in orange and the EAL domain in blue. Flavin and c-di-GMP are omitted for clarity.

SI References (main text numbering)

10. Barends, T. R. M., Hartmann, E., Griese, J. J., Beitlich, T., Kirienko, N. V., Ryjenkov, D. A., Reinstein, J., Shoeman, R. L., Gomelsky, M. & Schlichting, I. (2009). Structure and mechanism of a bacterial light-regulated cyclic nucleotide phosphodiesterase. *Nature* **459**, 1015-U150.

13. Tchigvintsev, A., Xu, X., Singer, A., Chang, C., Brown, G., Proudfoot, M., Cui, H., Flick, R., Anderson, W. F., Joachimiak, A., Galperin, M. Y., Savchenko, A. & Yakunin, A. F. (2010). Structural insight into the mechanism of c-di-GMP hydrolysis by EAL domain phosphodiesterases. *J Mol Biol* **402**, 524-38.

24. Minasov, G., Padavattan, S., Shuvalova, L., Brunzelle, J. S., Miller, D. J., Basle, A., Massa, C., Collart, F. R., Schirmer, T. & Anderson, W. F. (2009). Crystal structures of YkuI and its complex with second messenger cyclic Di-GMP suggest catalytic mechanism of phosphodiester bond cleavage by EAL domains. *J Biol Chem* **284**, 13174-84.

30. Winkler, A., Heintz, U., Lindner, R., Reinstein, J., Shoeman, R. L. & Schlichting, I. (2013). A ternary AppA–PpsR–DNA complex mediates light regulation of photosynthesis-related gene expression. *Nat Struct Mol Biol* **20**, 859-867.

39. Tarnawski, M., Barends, T. R., Hartmann, E. & Schlichting, I. (2013). Structures of the catalytic EAL domain of the Escherichia coli direct oxygen sensor. *Acta Crystallogr D Biol Crystallogr* **69**, 1045-53.

40. Chen, M. W., Kotaka, M., Vonrhein, C., Bricogne, G., Rao, F., Chuah, M. L., Svergun, D., Schneider, G., Liang, Z. X. & Lescar, J. (2012). Structural insights into the regulatory mechanism of the response regulator RocR from *Pseudomonas aeruginosa* in cyclic Di-GMP signaling. *J Bacteriol* **194**, 4837-46.

43. Jung, A., Domratcheva, T., Tarutina, M., Wu, Q., Ko, W. H., Shoeman, R. L., Gomelsky, M., Gardner, K. H. & Schlichting, I. (2005). Structure of a bacterial BLUF photoreceptor: insights into blue light-mediated signal transduction. *Proc Natl Acad Sci U S A* **102**, 12350-5.

44. Yuan, H., Anderson, S., Masuda, S., Dragnea, V., Moffat, K. & Bauer, C. (2006). Crystal structures of the Synechocystis photoreceptor Slr1694 reveal distinct structural states related to signaling. *Biochemistry* **45**, 12687-94.

52. Lindahl, E., Azuara, C., Koehl, P. & Delarue, M. (2006). NOMAD-Ref: visualization, deformation and refinement of macromolecular structures based on all-atom normal mode analysis. *Nucleic Acids Res* **34**, W52-6.

53. Svergun, D. I., Petoukhov, M. V. & Koch, M. H. (2001). Determination of domain structure of proteins from X-ray solution scattering. *Biophys J* **80**, 2946-53.

54. Petoukhov, M. V. & Svergun, D. I. (2005). Global rigid body modeling of macromolecular complexes against small-angle scattering data. *Biophys J* **89**, 1237-1250.

55. Ashkenazy, H., Erez, E., Martz, E., Pupko, T. & Ben-Tal, N. (2010). ConSurf 2010: calculating evolutionary conservation in sequence and structure of proteins and nucleic acids. *Nucleic Acids Res* **38**, W529-33.
