# Supplementary figures and images for: Characterization of Elements Involved in Allosteric Light Regulation of Phosphodiesterase Activity by Comparison of Different Functional BlrP1 States
Source: J Mol Biol. 2014 Feb 20;426(4):853–68. doi: 10.1016/j.jmb.2013.11.018 (PMC3989770; doi:10.1016/j.jmb.2013.11.018)

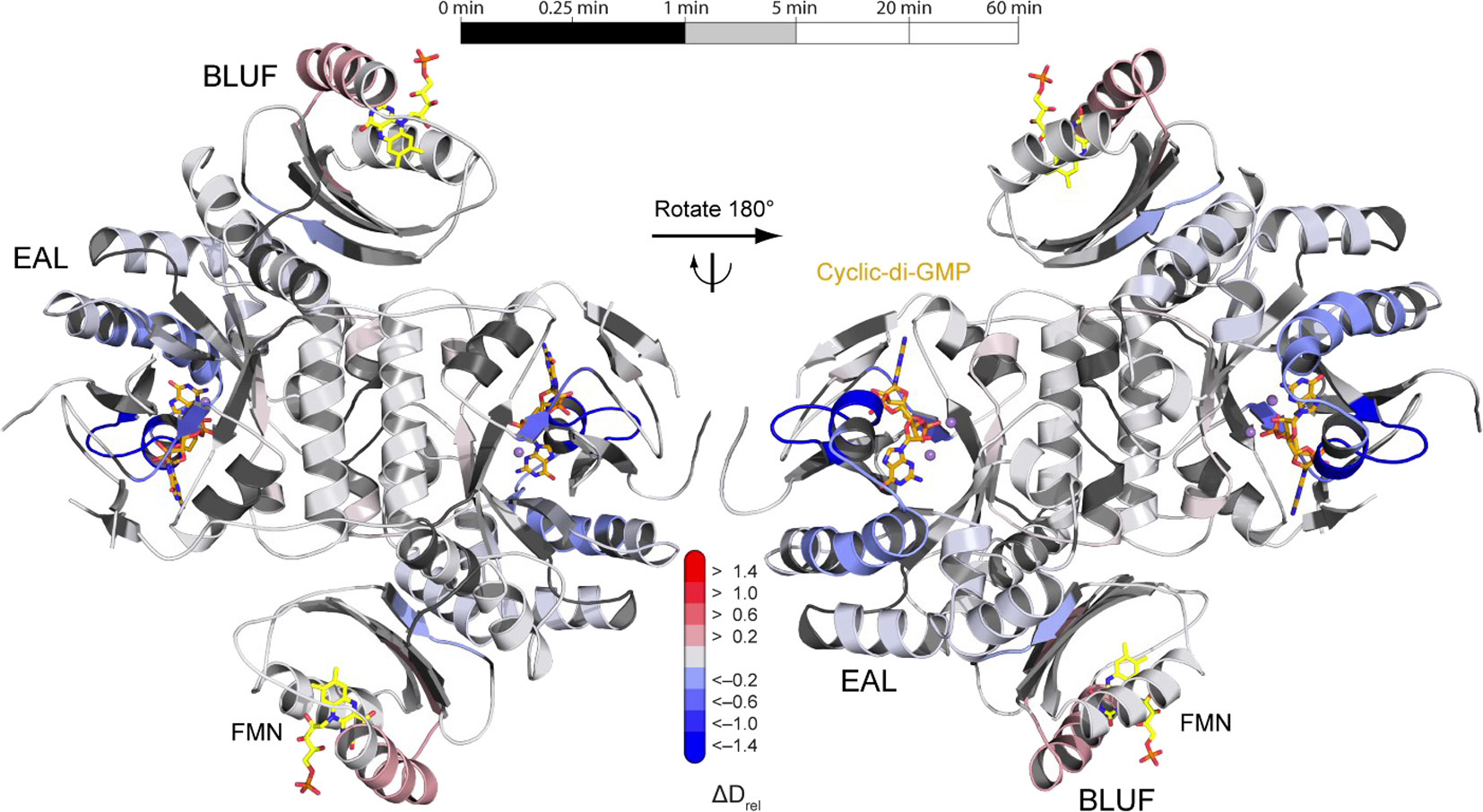

Supplement: Movie S1 — Animation of time-dependent changes in deuterium incorporation upon substrate and calcium binding in the dark. The time series of Ccd–Mgd comparisons is presented with colors corresponding to the differences in Drel according to the bar legend. Red or blue colors reflect an increased or decreased deuterium uptake, respectively, upon substrate coordination and calcium coordination. FMN and c-di-GMP are shown as yellow and orange stick models, respectively, and metal centers as purple spheres. Individual structural elements correspond to details of Fig. 3a. [file mmc2.jpg]

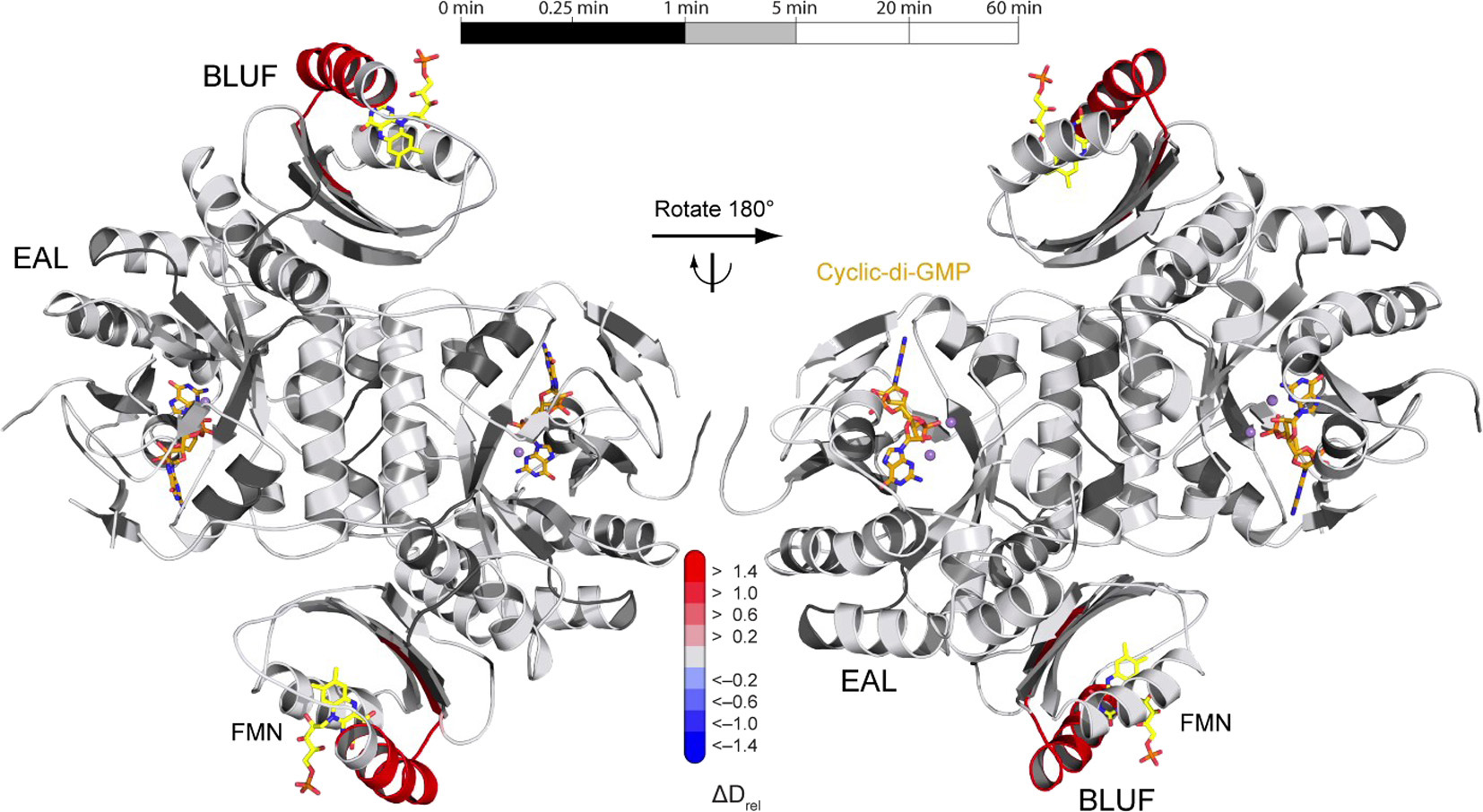

Supplement: Movie S2 — Animation of time-dependent changes in deuterium incorporation upon illumination in the presence of substrate and calcium. The time series of Ccl–Ccd comparisons is presented with colors corresponding to the differences in Drel according to the bar legend. Red or blue colors reflect an increased or decreased deuterium uptake, respectively, upon illumination in the presence of c-di-GMP and Ca2 +. FMN and c-di-GMP are shown as yellow and orange stick models, respectively, and metal centers as purple spheres. Individual structural elements correspond to details of Fig. 3b [file mmc3.jpg]

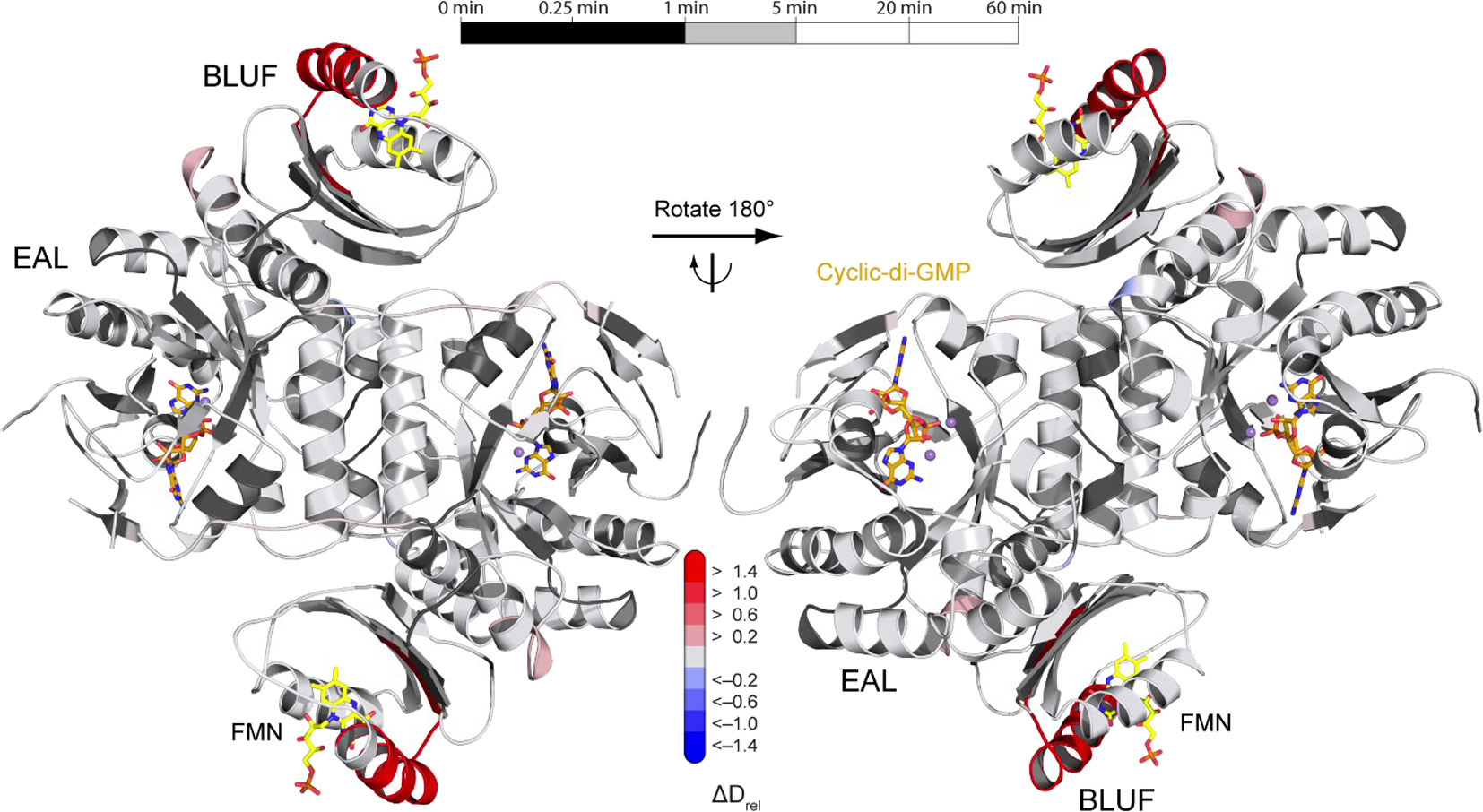

Supplement: Movie S3 — Animation of time-dependent changes in deuterium incorporation upon illumination in the absence of substrate and with Mg2 + present. The time series of Mgl–Mgd comparisons is presented with colors corresponding to the differences in Drel according to the bar legend. Red or blue colors reflect an increased or decreased deuterium uptake, respectively, upon illumination of BlrP1 in the presence of Mg2 +. FMN and c-di-GMP are shown as yellow and orange stick models, respectively, and metal centers as purple spheres. Individual structural elements correspond to details of Fig. 3c [file mmc4.jpg]

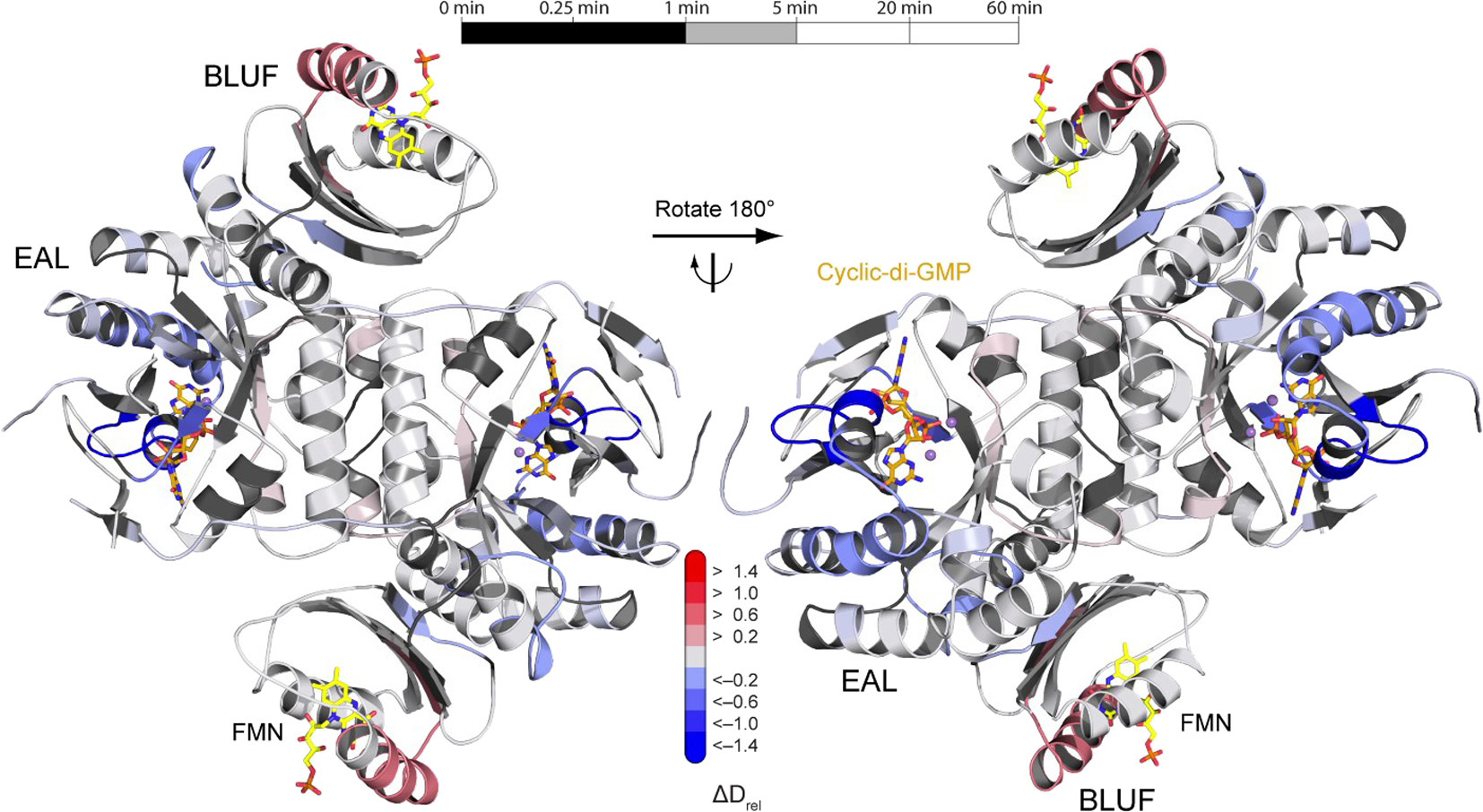

Supplement: Movie S4 — Animation of time-dependent changes in deuterium incorporation upon substrate and calcium binding in light-adapted BlrP1. The time series of Ccl–Mgl comparisons is presented with colors corresponding to the differences in Drel according to the bar legend. Red or blue colors reflect an increased or decreased deuterium uptake, respectively, upon substrate coordination and calcium coordination in the light-adapted BlrP1 state. FMN and c-di-GMP are shown as yellow and orange stick models, respectively, and metal centers as purple spheres. Individual structural elements correspond to details of Fig. 3d. [file mmc5.jpg]

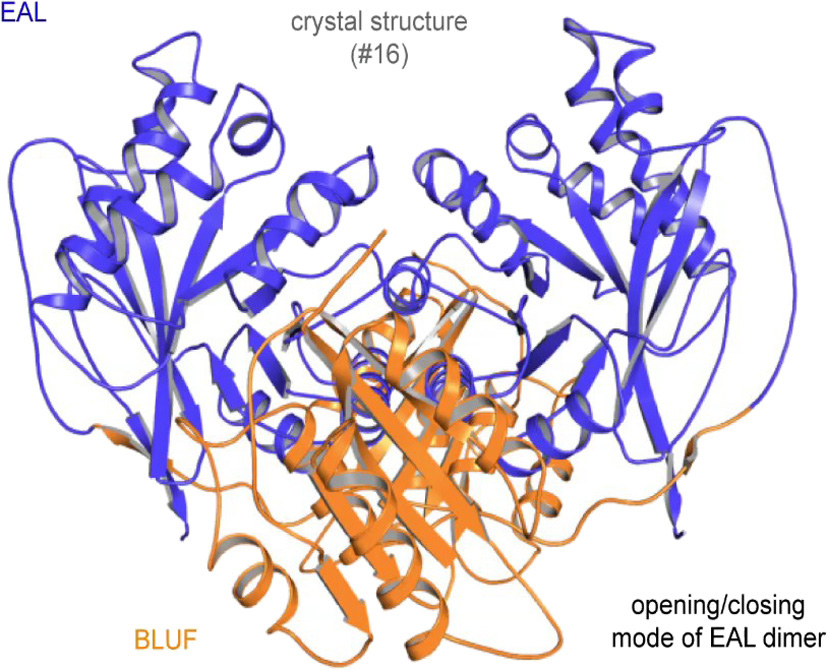

Supplement: Movie S5 — Animation of the normal mode best describing the differences between experimental SAXS data and the crystal structure. Thirty substructures of normal mode 8 (NOMAD-Ref output) with the maximal amplitude between structures #8 and #23 are shown as cartoon representation. The BLUF domain is colored orange and the EAL domain is in blue. Flavin and c-di-GMP are omitted for clarity. [file mmc6.jpg]

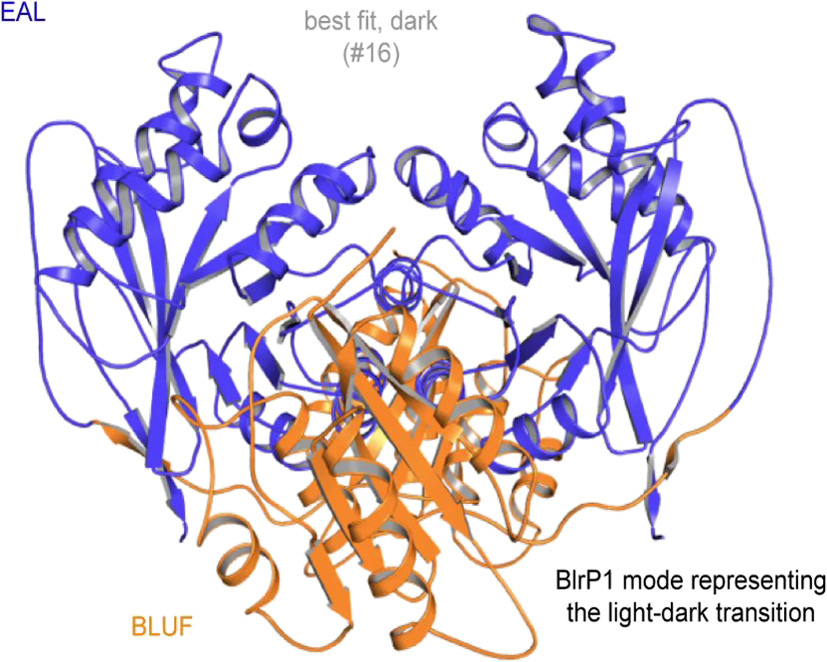

Supplement: Movie S6 — Animation of the normal mode representing the light-induced structural changes observed for BlrP1. Thirty substructures corresponding to one full cycle of normal mode 10 (NOMAD-Ref output) are shown as cartoon representation. The BLUF domain is colored orange and the EAL domain is in blue. Flavin and c-di-GMP are omitted for clarity. [file mmc7.jpg]
